# Cardiac radiotherapy–induced epigenetic memory underlies electrophysiologic and metabolic reprogramming

**DOI:** 10.1172/JCI193087

**Published:** 2026-02-17

**Authors:** Samuel D. Jordan, Shuhua Fu, Abigail Fulkerson, Donghua Hu, Sherwin Ng, David M. Zhang, Sneha Manikandan, Jeffrey Szymanski, Nan Hu, Yuqian Xie, Anish Bedi, James Tabor, Lauren Boggs-Bailey, Lori Strong, Stephanie Hicks, Lavanya Aryan, Nishanth Gabriel, Geoffrey D. Hugo, Kuo-Chan Weng, Nathaniel Huebsch, Julie K. Schwarz, Bo Zhang, Stacey L. Rentschler

**Affiliations:** 1 Department of Internal Medicine, Cardiovascular Division,; 2 Center for Noninvasive Cardiac Radiotherapy,; 3 Department of Developmental Biology, and; 4 Department of Radiation Oncology, Washington University, School of Medicine, St. Louis, Missouri, USA.; 5 Department of Radiation Oncology, Mayo Clinic, Rochester, Minnesota, USA.; 6 Mayo Clinic Comprehensive Cancer Center, Rochester, Minnesota, USA.; 7 Center of Regenerative Medicine,; 8 Department of Biomedical Engineering,; 9 Alvin J. Siteman Cancer Center,; 10 Department of Cell Biology and Physiology, Washington University, School of Medicine, St. Louis, USA.

**Keywords:** Cardiology, Cell biology, Arrhythmias, Epigenetics, Radiation therapy

## Abstract

Stereotactic arrhythmia radiotherapy (STAR) is emerging as a highly effective treatment for ventricular tachycardia (VT). Growing evidence indicates that STAR favorably reprograms the electrical substrate by speeding conduction and/or prolonging repolarization via modulation of ion channel expression, although the mechanisms by which single-fraction radiation mediates durable changes in gene expression are incompletely understood. Here, we identify dynamic changes in the cardiomyocyte epigenome and transcriptome after irradiation (IR) in vivo and in vitro, including durably increased expression and chromatin accessibility of *Scn5a* (encodes the α subunit of the sodium channel, Na_V_1.5), demonstrating a role for epigenetic memory in conduction velocity (CV) increases observed after STAR. Transcriptomic and epigenetic sequencing further identified dynamic changes in gene expression and regulatory regions involved in cellular repolarization, calcium handling, and metabolism after IR. These changes were mirrored by dose-dependent and cell-autonomous changes in repolarization, calcium flux, and mitochondrial respiration, highlighting important cellular processes that may mediate the therapeutic effects of STAR. Overall, we found that cardiomyocytes exposed to a single fraction of high-dose IR exhibited epigenetic reprogramming that mediated broad and dynamic physiologic responses.

## Abstract 2

Radiation mediates durable changes to the cardiomyocyte transcriptome and epigenome with associated electrophysiologic reprogramming that may underlie the antiarrhythmic effect of stereotactic arrhythmia radiotherapy.

## Graphical abstract

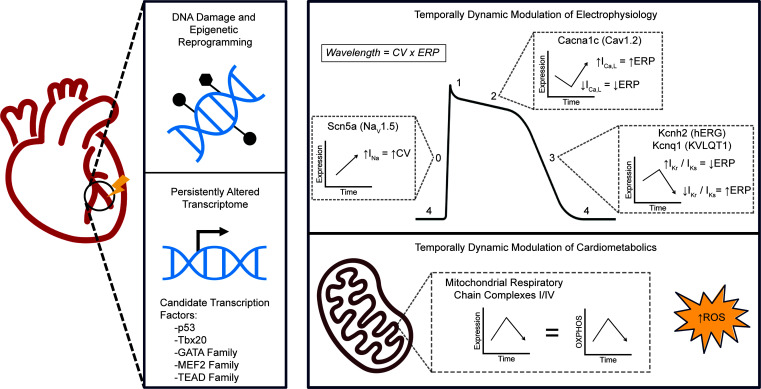

## Introduction

Ventricular tachycardia (VT) is an arrhythmia associated with sudden cardiac death, a leading cause of mortality (1). Existing therapies are limited: antiarrhythmic drugs are associated with toxicity, and implantable cardioverter defibrillator shocks affect quality of life (2–4). Catheter ablation offers definitive treatment by replacing VT circuits with an electrically inert scar, but over half of patients experience recurrence (5), making novel treatments of great clinical interest. Stereotactic arrhythmia radiotherapy (STAR) has emerged as a highly effective intervention for refractory VT, demonstrating a 94% reduction in VT episodes over the 6-month period after a single dose of 25 Gy in a phase I/II trial, with reductions persisting to at least 12 months (6, 7). Subsequent trials have replicated similarly remarkable results (8–14), supporting the therapeutic potential of STAR, which is now undergoing a phase III clinical trial (NCT05765175).

STAR’s antiarrhythmic mechanism remains incompletely understood. VT most commonly arises from damaged myocardium, where slowed conduction through an abnormal electrical substrate promotes reentry (1, 2). Preclinical data have demonstrated that doses of more than 40 Gy can induce fibrosis and atrioventricular (AV) block (14); however, the clinically used dose for STAR (25 Gy) does not induce substantial fibrosis in animal models or patients (15–17). Additionally, rapid clinical responses are inconsistent with the timing of irradiation-induced (IR-induced) fibrosis (6, 7). Instead, emerging evidence demonstrates that STAR favorably alters the electrical substrate to decrease the likelihood of sustained reentry. Reentry requires that the conduction wavelength (the distance traveled by an electrical impulse during the myocardial excitable gap) is less than the pathlength of the circuit. Speeding conduction and/or prolonging the refractory period increases the wavelength, thereby terminating reentry (2).

Our group and others have demonstrated that IR leads to long-lasting upregulation of ventricular gap-junction protein connexin 43 (Cx43) and Na_V_1.5 expression coincident with long-term conduction velocity (CV) increases in mice and humans (15, 18–20). More recently, we demonstrated that STAR locally prolongs the refractory period in a porcine model of VT (16). Clinically, reductions in VT are durable for months to years, and recurrences typically exhibit new VT morphologies originating from outside the planning target volume (PTV) that received the prescribed dose of approximately 25 Gy, suggesting persistent suppression of the original VT circuit (13, 21). These results demonstrate durable cardiac electrical reprogramming in response to STAR, potentially explaining its efficacy in treating VT; however, the mechanism by which STAR persistently alters ion channel expression remains incompletely understood.

At the molecular level, IR triggers cellular responses directly through interaction with DNA molecules causing DNA damage, or indirectly via the transfer of energy to other molecules, especially water, to generate ROS (22). This triggers a cascade of cellular responses, known as the DNA damage response (DDR), to either repair the damaged DNA or trigger cell death (22, 23). This work has focused on mitotically active cells, so the response of terminally differentiated cells (including cardiomyocytes) is less well characterized. Epigenetic regulators are recruited as part of the DDR, leading to acute changes to epigenetic modifications and the chromatin state (23, 24). The long-term effect of this remodeling is unclear: chromatin may be restored to its initial state, or permanent changes may create epigenetic memory of DNA-damaging events (23), which could contribute to persistently altered transcriptional states after IR.

We hypothesized that radiation mediates antiarrhythmic electrophysiologic reprogramming via epigenetic remodeling in cardiomyocytes. We first identified persistent structural changes to the cardiomyocyte nucleus after IR through histopathologic assessment, which we speculate is indirect evidence of epigenetic remodeling due to the link between chromatin structure and nuclear morphology. Next, we profiled the transcriptome and epigenome of cardiomyocytes after IR, both in vivo and in vitro. This demonstrated temporally dynamic transcriptional and epigenetic changes after IR, including transient changes at acute time points, durable changes at late time points, and bidirectional responses–genes or chromatin regions that changed in one direction acutely and the other direction at late time points — underscoring the complex evolution of the molecular responses of cardiomyocytes to IR. We observed durable chromatin remodeling around *Scn5a* (encodes the α subunit of the sodium channel, Na_V_1.5), implicating post-IR epigenetic memory in the antiarrhythmic effects of STAR. Our multimodal sequencing approach identified transcriptomic reprogramming of additional, previously underexplored aspects of cardiomyocyte biology that may be important to STAR’s therapeutic effects. Physiologic characterization of human induced pluripotent stem cell–derived cardiomyocytes (hiPSC-CMs) demonstrated dose-dependent and cardiomyocyte-autonomous responses to IR corresponding to the observed transcriptional and epigenetic changes, which included not only expected increases in CV, but also dynamic modulation of the effective refractory period (ERP), calcium flux, and oxidative phosphorylation (OXPHOS). Overall, we show that epigenetic remodeling underlies diverse, dynamic physiologic responses in cardiomyocytes after IR, which likely mediate the antiarrhythmic effects of STAR and suggest dose-dependent toxicities.

## Results

### High-dose IR alters cardiomyocyte nucleus size without changes to ploidy or cell size.

**Figure 1:**
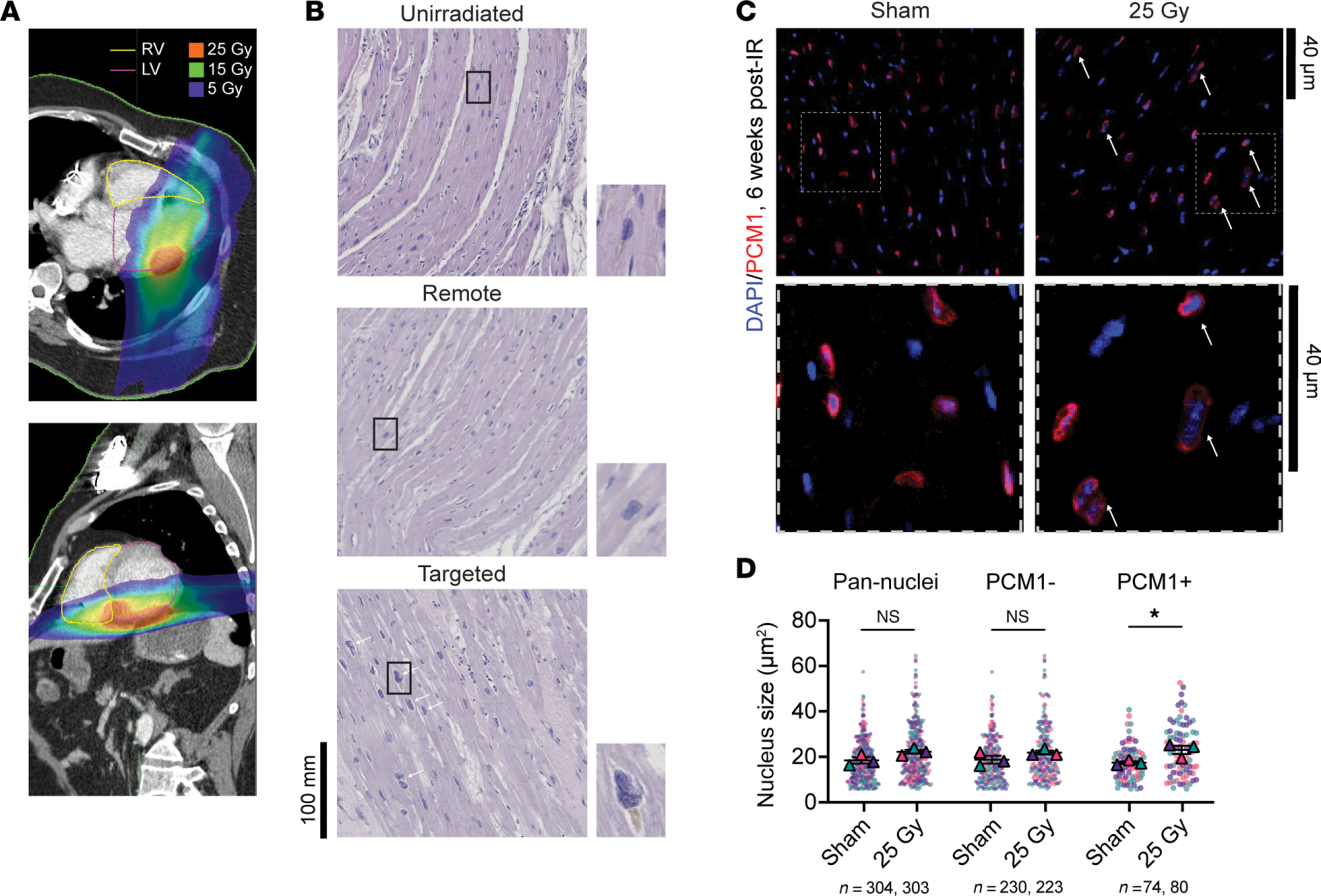
Cardiac radiation alters cardiomyocyte nucleus size. (**A**) Thoracic CT scan images used for radiation treatment planning for a patient receiving STAR. Color wash shows the radiation dose distribution. Yellow outline indicates the right ventricle (RV); purple outline indicates the LV. (**B**) H&E staining of targeted and remote regions of an irradiated heart 209 days after STAR compared with an unirradiated, nonfailing donor heart. Scale bar: 100 mm. Nuclear morphology is highlighted in the magnified regions. Original magnification, ×20; insets: original magnification ×60. (**C**) Representative PCM1 (red) and DAPI (blue) immunostaining of sham-treated versus irradiated mouse hearts 6 weeks after treatment. White arrows indicated nuclei with double-positive staining. Scale bars: 40 μm. (**D**) Nuclear cross-sectional areas in control versus irradiated hearts 6 weeks after treatment by DAPI fluorescence subdivided into pan-nuclei (*P*_adj_ = 0.17), PCM1^–^ nuclei (*P*_adj_ = 0.31), and PCM1^+^ cardiomyocyte nuclei (*P_adj_ = 0.031) (2-way ANOVA, Šídák’s post hoc test, *P*_treatment_ = 0.002, *P*_celltype_ = 0.99, *P*_interaction_ = 0.63). Colors represent different mice.

A typical STAR treatment plan uses a prescribed dose of 25 Gy delivered to the periphery of the PTV with a higher dose inside the PTV and a wide range of dose deposition throughout the heart (Figure 1A). Nuclear atypia — defined by “smudgy” chromatin and irregular nuclear membranes as well as increased nucleus size — are persistent histopathologic hallmarks of prior radiation exposure across human tissues (25). We therefore assessed for changes to nuclear morphology in both targeted (≥25 Gy) and untargeted (<5 Gy) regions of a human heart explanted 209 days after STAR. H&E-stained tissue sections demonstrated frequent large nuclei with atypia in targeted regions of the irradiated heart, but we rarely observed these nuclear changes in untargeted regions, and not at all in an unirradiated donor heart (Figure 1B).

To further explore these nuclear changes, we treated mice with 25 Gy whole-heart IR and analyzed 6 weeks later. Pericentriolar material 1 (PCM1) immunofluorescence was used to differentiate cardiomyocytes from noncardiomyocytes (26). The left ventricles (LVs) of irradiated mice had significantly increased nuclei in PCM1^+^ cells (cardiomyocytes), but not in PCM1^–^ cells (noncardiomyocytes) (Figure 1, C and D). Nuclear morphology was determined by factors including ploidy, nuclear transport and envelope proteins, cytoskeletal architecture, and chromatin state (27). Adult mammalian cardiomyocyte ploidy increases after injury (28); however, PCM1- and DAPI-based fluorescence-activated nuclei sorting (FANS) (29) revealed no difference in cardiomyocyte nuclear ploidy between sham-treated and irradiated mouse hearts (Supplemental Figure 1, A and B; supplemental material available online with this article; https://doi.org/10.1172/JCI193087DS1). An increase nucleus size could also reflect rising transcriptional demands due to cellular hypertrophy, but cardiomyocyte cell length was unchanged (Supplemental Figure 1, C and D), consistent with our previous findings (15).

### Cardiac radiation dynamically alters chromatin accessibility and gene expression in murine cardiomyocytes.

**Figure 2:**
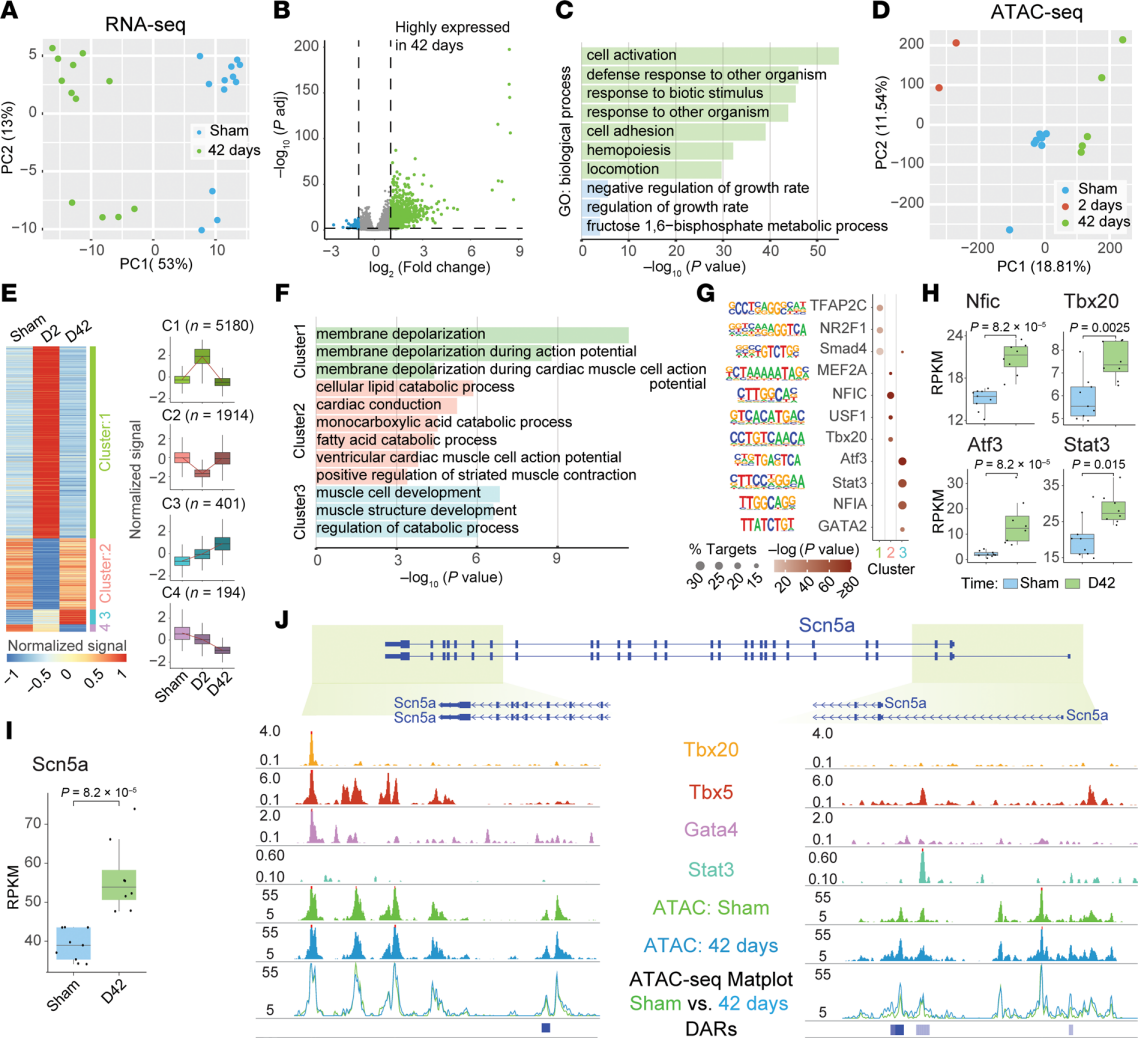
Cardiac radiation induces dynamic changes in gene expression and chromatin accessibility in murine PCM1^+^ nuclei. (**A**) PCA for sham (green) and 42 days after IR (blue) mouse PCM1^+^ nuclei transcriptomes. (**B**) Volcano plot of significantly upregulated (green) and downregulated (blue) transcripts in day-42 post-IR–treated versus sham-treated murine cardiomyocyte nuclei. (**C**) Selected GO terms enriched in upregulated (green) and downregulated (blue) genes. (**D**) PCA of ATAC-seq samples for PCM1^+^ nuclei from sham-treated, day-2 (D2) post-IR, and day-42 (D42) post-IR mouse hearts. (**E**) Heatmap of DARs in PCM1^+^ nuclei over time after 25 Gy compared with sham, organized by *k*-means clustering. (**F**) Selected GO term analysis for *k*-means clustered DARs for clusters 1–3. (**G**) HOMER motif analysis of TF binding motifs enriched in each cluster of DARs. (**H**) mRNA transcript levels of selected TFs identified in motif enrichment analysis (**H**) and *Scn5a* (**I**) in sham-treated nuclei versus day-42 post-IR PCM1^+^ nuclei (*P* values were determined by Wilcoxon ranked-sum test). (**J**) Genome browser plot of the *Scn5a* gene showing sham and day-42 post-IR ATAC-seq tracings, as well as ChIP-seq chromatin occupancy for Tbx20 (orange), Tbx5 (red), *GATA4* (purple), and Stat3 (green). Significant DARs are highlighted below the ATAC-seq Matplots. Insets highlight the TSS (left) and known enhancer elements (left).

Recent studies highlight the interplay between epigenetic state and nuclear morphology, mediated by chromatin-lamin-cytoskeleton interactions (27). Since nucleus enlargement after IR was not associated with increased chromatin content, we hypothesized that 25 Gy may instead persistently alter the cardiomyocyte chromatin state. To test this hypothesis, we performed RNA-seq (sham vs. 42 days after IR) and assayed for transposase-accessible chromatin sequencing (ATAC-seq) (sham vs. 2 and 42 days after IR) on PCM1-sorted cardiomyocyte nuclei from mouse LVs. Principal component analysis (PCA) revealed distinct transcriptional states of sham-treated and day-42 post-IR cardiomyocytes (Figure 2A and Supplemental Table 1). Differential expression analysis identified 808 differentially expressed genes (DEGs), the majority of which (*n* = 740) were upregulated after IR (Figure 2B). Gene ontology (GO) analysis revealed upregulation of genes involved in cell activation and cellular defense with downregulation of growth and metabolic genes (Figure 2C and Supplemental Table 2), likely representing stress responses to IR.

PCA of ATAC-seq data demonstrated the most dramatic differences relative to sham treatment 2 days after IR, with more modest differences persisting 42 days after IR, highlighting the dynamic changes in chromatin accessibility over time (Figure 2D and Supplemental Table 3). We identified 7,689 chromatin regions as differentially accessible across time. *K*-means clustering identified 4 clusters of differentially accessible regions (DARs), defined by transiently increased accessibility (cluster 1; *n* = 5,180 DARs), transiently decreased accessibility (cluster 2; *n* = 1,914 DARs), persistently increased accessibility (cluster 3; *n* = 401 DARs), and persistently decreased accessibility (cluster 4; *n* = 194 DARs) (Figure 2E). Although many DARs changed acutely and then resolved (clusters 1 and 2), a fraction of DARs (*n* = 595; clusters 3 and 4) retained durably altered chromatin states after one-time treatment with IR. GO analysis revealed genes near DARs involved in membrane depolarization in cluster 1; in cardiac conduction, contraction, and metabolic processes in cluster 2; and in muscle development and regulation of catabolic processes in cluster 3 (Figure 2F and Supplemental Table 4). Enhancer regions represented approximately 20% of DARs, and approximately 12% were located in polycomb-enriched regions (ReprPC and TSSBiv), with a smaller fraction located at transcription start sites (TSSs) (9%) and other genomic regions (Supplemental Figure 2).

Motif enrichment analysis identified transcription factor (TF) binding motifs enriched in DAR clusters (Figure 2G). Four identified TFs were also significantly transcriptionally upregulated: nuclear factor 1C (*Nfic*), T box transcription factor 20 (*Tbx20*), activating transcription factor 3 (*Atf3*), and *Stat3* (Figure 2H). Transcription of *Scn5a*, which encodes Na_V_1.5, increased significantly after IR (Figure 2I), consistent with our previous studies demonstrating increased Na_V_1.5 protein after IR (15). We noticed increased chromatin accessibility around the *Scn5a* TSS, along with co-occupancy of *Stat3* and *Tbx5*, as well as known downstream enhancer regions bound by *Tbx20*, *Tbx5*, and *Gata4* (Figure 2J) (30). These findings implicate chromatin dynamics in the beneficial electrophysiologic reprogramming that appears to confer STAR’s antiarrhythmic effect.

**Figure 3:**
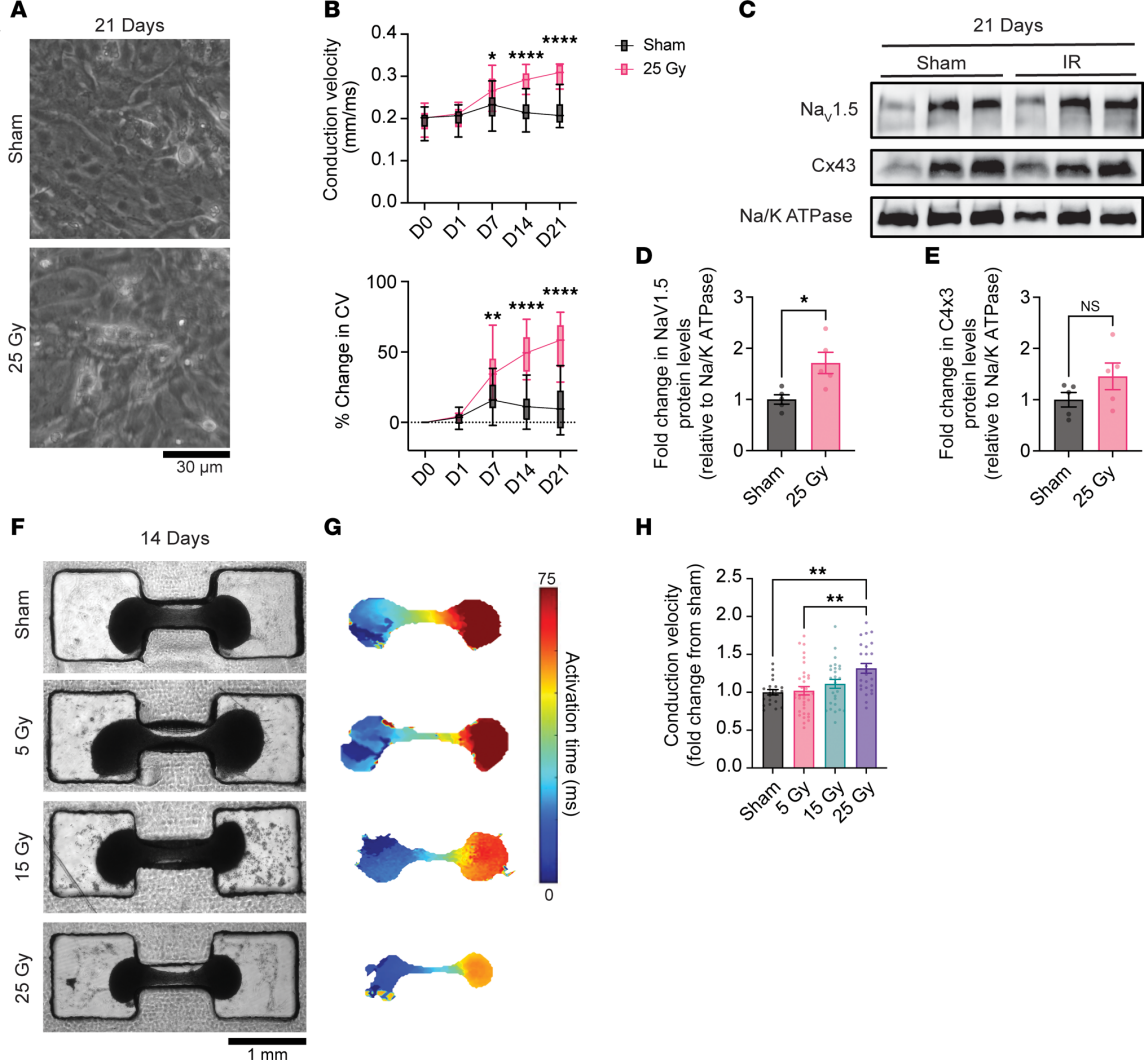
IR induces cardiomyocyte-autonomous CV increases in a dose-dependent fashion in hiPSC-CM models. (**A**) Tissue culture images of sham-treated and irradiated hiPSC-CMs 3 weeks after IR. Scale bar: 30 μm. (**B**) CV measurements assessed by MEA over time, shown as the CV and percentage of change in CV from day 0. Boxes represent the 25th–75th percentiles, and whiskers represent minimum-to-maximum values (*n*_wells_ = 24 sham, 12 irradiated from 3 cryovials; 2-way ANOVA: *P*_time,_ *P*_treatment_, and *P*_interaction_ < 0.0001; Šídák’s post hoc test: *P_adj_ = 0.030, **P_adj_ = 0.005, ****P_adj_ < 0.0001). (**C**) Western blots probed for Na_V_1.5, Cx43, and Na/K ATPase (loading control) from membrane preparations from sham-treated versus 25 Gy irradiated hiPSC-CMs 21 days after treatment. (**D** and **E**) FC in quantified density of Na_V_1.5 (**D**) and Cx43 (**E**) (*n* = 5 cryovials; 2-tailed *t* test: *P_NaV1.5_ = 0.014, *P*_Cx43_ = 0.16). (**F**) Representative tissue culture images of sham-treated and 5, 15, and 25 Gy irradiated μEHTs 14 days after treatment. Scale bar: 1 mm. (**G**) Representative activation maps from optocardiography of sham-treated and 5, 15, and 25 Gy irradiated μEHTs 14 days after treatment. Tissues were stimulated at 1 Hz from the left knob. (**H**) Quantified CV in sham-treated versus 5, 15, and 25 Gy irradiated μEHTs 14 days after treatment, presented as the FC relative to the sham average by batch (≥22 μEHTs per condition from 5 differentiations; 1-way ANOVA: *P* = 0.0006; Tukey’s post hoc test: **P_adj,sham–25Gy_ = 0.002, **P_adj,5Gy–25Gy_ = 0.001).

*25 Gy IR increases CV and upregulates Na_V_1.5 in a cardiomyocyte-autonomous fashion*. Radiation can trigger secondary effects (e.g., microvascular ischemia) and initiate cell-to-cell signaling and bystander responses, whereby nonirradiated cells are influenced via the release of soluble factors from irradiated cells (22, 31). To assess whether IR-mediated cardiomyocyte reprogramming is cell autonomous (intrinsic to the cardiomyocyte) or non-cell-autonomous (dependent on other cell types), we utilized hiPSC-CMs to test the response of isolated cardiomyocytes in vitro. iCell Cardiomyocytes^2^ (iCells) (FujiFilm; female donor) were cultured on a multielectrode array (MEA) and exposed to 25 Gy or sham treatment, followed by serial electrophysiologic assessment. Sham and irradiated cells remained viable with the appropriate morphology for at least 3 weeks (Figure 3A), with increased nucleus size after IR, as we saw in mice (Supplemental Figure 3, A and B). Both sham-treated and irradiated cells demonstrated increasing CV over the 3-week period, consistent with cellular maturation. A greater increase was seen in irradiated cells relative to sham-treated cells as early as day 7, persisting to day 21, at which time the irradiated cells demonstrated a greater absolute CV (0.30 ± 0.007 mm/ms versus 0.22 ± 0.006 mm/ms; *P* ≤ 0.0001) and percentage increase from day 0 (56% ± 4.8% versus 10% ± 3.1%; *P* ≤ 0.0001; Figure 3B).

We next assessed protein levels of ion channels previously shown to be upregulated in vivo (15). Membrane fractionation and Western blotting performed on day 21 revealed a 1.71-fold increase in Na_V_1.5 versus sham treatment (SEM 0.21, *P* = 0.01; Figure 3, C and D). Although Cx43 expression showed an upward trend, the increases were too variable to reach statistical significance (fold change [FC] = 1.46 ± 0.26, *P* = 0.16; Figure 3, C and E).

### IR of engineered human heart tissues increases CV in a dose-dependent fashion.

Human iPSC-CMs are immature and characterized by fetal transcriptional profiles, disorganized morphology, glycolytic metabolism, and immature action potential morphologies (32). Microengineered heart tissues (μEHTs) are a tissue-engineered model (90% hiPSC-CMs, 10% fibroblasts) that applies chronic prestress to mature hiPSC-CMs (33, 34). We generated μEHTs using a second iPSC line (WTC, Coriell Repository no. GM2525, male donor) to test whether more mature hiPSC-CMs respond similarly to IR and to rule out cell line–, differentiation method–, and sex-specific responses (35). Although 25 Gy is the commonly prescribed dose delivered to the PTV boundary, other areas of the heart receive widely variable doses (Figure 1A), and the optimal dosing strategy for STAR’s antiarrhythmic effect is unknown. We therefore treated μEHTs with 25, 15, or 5 Gy versus sham and performed optical mapping 2 weeks later. Tissues treated with 25 Gy exhibited significantly faster CV than did sham or a 5 Gy dose (FC relative to sham = 1.32 ± 0.06; *P* = 0.002), while there was a nonsignificant 1.11-fold increase over sham after 15 Gy (SEM 0.06; *P* = 0.55; Figure 3, G and H). CV increased as early as 7 days after 25 Gy (*P* = <0.0001; Supplemental Figure 5, B and C), consistent with the timing observed in MEA studies. Conduction changes were independent of changes in μEHT size or structure (Figure 3F, Supplemental Figure 4, and Supplemental Figure 5A).

### CV increases are not explained by cellular hypertrophy or fibroblast reduction in μEHTs.

Increased cardiomyocyte cell size can increase cell capacitance, allowing more rapid current flow through low-resistance cytoplasm (36). Cardiomyocyte length and width were unchanged in μEHTs after IR (Supplemental Figure 6, A–C), consistent with our murine findings above. Changes to the cellular composition of μEHTs, such as the fibroblast percentage, could also influence CV after IR (36), but immunostaining for the fibroblast marker vimentin showed no change in the percentage of area occupied by fibroblasts (Supplemental Figure 6, D and E). Therefore, changes in cardiomyocyte cell size or fibroblast numbers were unlikely to have contributed to the IR-mediated increased CV in μEHTs.

### 25 Gy regulates gene expression and the chromatin landscape in hiPSC-CMs.

**Figure 4:**
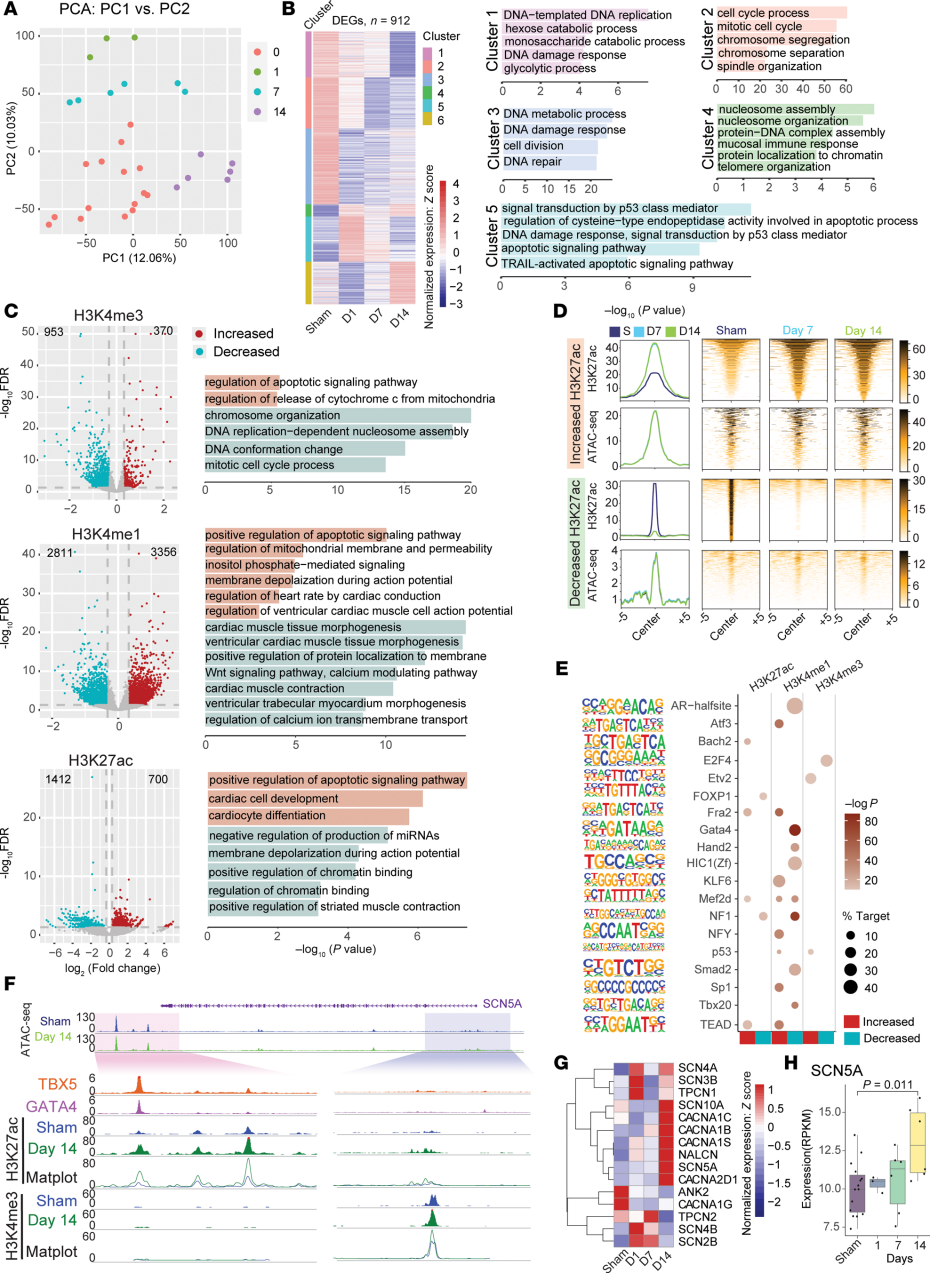
25 Gy IR alters the epigenetic landscape of hiPSC-CMs. (**A**) PCA of RNA-seq samples for sham-treated and day-1, -7, and -14 post-IR time points in iCells. (**B**) Heatmap of DEGs in iCells over time after 25 Gy IR, organized by *k*-means clustering with GO term enrichment by cluster. (**C**) Volcano plots for genomic regions with increased (red) and decreased (blue) reads from CUT&Tag experiments targeting H3K4me3, H3K4me1, and H3K27ac, with GO term enrichment. (**D**) ATAC-seq and H3K27ac Matplots and heatmaps in sham-treated (dark blue), day-7 IR-treated (light blue), and day-14 IR-treated (light green) hiPSC-CMs for increased and decreased differentially acetylated regions. (**E**) HOMER Motif analysis of TF binding motifs enriched in DMRs. (**F**) Genome browser plot of the *SCN5A* locus showing changes in the epigenetic landscape after 25 Gy IR. (**G**) Heatmap of RNA expression for genes in the GO term “Membrane depolarization during action potential.” (**H**) mRNA *CN5A* transcript levels from RNA-seq (*P* values were determined by Wilcoxon ranked-sum test).

To further understand the dynamic molecular responses of human cardiomyocytes to radiation, we performed a multiomics sequencing assay across time in both iCell (RNA-seq + ATAC-seq at 1, 7, and 14 days; Cleavage Under Targets and Tagmentation [CUT&Tag] at 7 and 14 days) and WTC (RNA-seq + ATAC-seq at 1, 7, 14, and 28 days) hiPSC-CMs cultured in adherent 2D monolayers. In both cell lines, PCA showed an alteratuib of the transcriptome and epigenome that was most pronounced at 1 day after IR, followed by a gradual trend back toward the sham condition, although irradiated cells remained distinct throughout (Figure 4A, Supplemental Figure 7A, and Supplemental Figure 8, A and C). We identified 912 DEGs in iCells and 1,128 DEGs in WTC hiPSC-CMs across time after IR. GO analysis after *k*-means clustering revealed that acutely upregulated DEGs (cluster 5 in both cell lines) were enriched for the p53 pathway and DNA damage responses, with a corresponding downregulation of transcripts involved in cell cycling and proliferation (iCell clusters 1–3; WTC clusters 2 and 3) (Figure 4B, Supplemental Figure 8B, and Supplemental Tables 5–8). As in mice, many acute changes in chromatin accessibility resolved with time (iCell clusters 1, 3, and 5; WTC clusters 3 and 4), but other regions of the genome displayed persistently decreased (iCell cluster 2; WTC cluster 2) or increased (iCell clusters 4 and 6; WTC cluster 1) accessibility (Supplemental Figure 7B, Supplemental Figure 8D, and Supplemental Tables 9 and 10). GO analysis revealed enrichment of genes involved in cardiac conduction and contraction in DARs (iCell clusters 1 and 3, WTC cluster 2; Supplemental Figure 7B, Supplemental Figure 8D, and Supplemental Tables 11 and 12).

To further characterize changes in the epigenetic landscape after 25 Gy IR, we performed CUT&Tag in iCells for the histone marks H3K27ac, H3K4me1, H3K4me3, and H3K27me3 at post-IR days 7 and 14 and compared them with sham cells. In total, we identified 1,323 differentially modified regions (DMRs) for H3K4me3, a marker of transcription at the TSS (370 decreasing, 953 increasing); 6,167 DMRs for H3K4me1, a marker of enhancers (3,356 decreasing, 2,811 increasing); 2,112 DMRs for H3K27ac, an activating marker at enhancers and promoters (700 decreasing, 1,412 increasing); and 2,144 DMRs for H3K27me3, a repressive marker (832 decreasing, 1,312 increasing) (Figure 4C, Supplemental Figure 7C, and Supplemental Table 13) (37). Particularly notable were the extensive changes at enhancer regions (H3K4me1 and H3K27ac).

GO analysis demonstrated that genes associated with increased H3K4me3, H3K27ac, and H3K4me1 modifications were enriched in the apoptosis pathway, whereas genes associated with decreased H3K4me3 modifications were enriched in DNA replication and mitosis pathways (Figure 4C and Supplemental Table 14). These changes in epigenetic markers of active promoters and enhancers were consistent with the reduced active transcription of cell-cycle genes due to p53 activation observed with RNA-seq. Terms associated with cardiomyocyte development and membrane depolarization were enriched among H3K27ac DMRs (Figure 4C). GO terms associated with cardiomyocyte biology were enriched among H3K4me1 DMRs, including increased H3K4me1 near genes involved in cardiac conduction and mitochondrial function and decreased H3K4me1 near genes involved in cardiac tissue morphogenesis, contraction, and calcium handling (Figure 4C), illustrating the importance of enhancer remodeling in post-IR cardiomyocyte physiology.

We compared DARs identified by ATAC-seq with histone modification CUT&Tag data. Many DARs overlapped with H3K4me1 (21%), H3K4me1 plus H3K27ac (23%), or H3K4me1 plus other marks, for a total of 47% of DARs overlapping with H3K4me1 (Supplemental Figure 7D). This further suggests changes in chromatin accessibility at enhancer regions after IR, which is consistent with the chromatin states analysis in mice (Supplemental Figure 2). H3K27ac DMRs showed no detectable changes in chromatin accessibility over time (Figure 4D). Additionally, regions with increased H3K27ac after IR were more accessible across conditions than were those with decreased H3K27ac after IR (Figure 4D). This suggests that these histone modifications occurred within a preestablished chromatin accessibility landscape, and radiation appeared to reactivate these enhancers.

Histone modifications both influence and are influenced by the recruitment and binding of TFs. Therefore, we probed for enrichment of TF binding motifs in regions with differential histone modifications, which revealed enrichment of TBX20 binding motifs in regions with decreased H3K4me1, as observed for murine DARs, further suggesting a role for TBX20 in post-IR gene regulation.

We next asked whether post-IR epigenetic reprogramming in hiPSC-CMs may contribute to the cell-autonomous upregulation of Na_V_1.5. Unlike in murine cardiomyocytes, we did not detect significant changes in chromatin accessibility near the TSS of *SCN5A* (Figure 4F). This discrepancy could reflect a non-cell-autonomous response but may also be due to the relatively early time point in hiPSC-CMs (day 14 vs. day 42 in mice). We observed other DARs associated with *SCN5A* (cluster 1, Supplemental Figure 7B), demonstrating dynamic chromatin remodeling near the *SCN5A* locus. It is possible that this remodeling would continue and result in a significantly more accessible state at a later time point, as observed in mice. We detected substantial changes in active markers H3K27ac and H3K4me3 associated with the *SCN5A* locus, both at the TSS and downstream enhancers (Figure 4F). We also observed late transcriptional upregulation of numerous genes in the GO term “membrane depolarization during action potential,” including a significant increase in *SCN5A* (Figure 4, G and H). Overall, these findings demonstrate cell-autonomous transcriptional and epigenetic remodeling in response to 25 Gy, implicating genes involved in cardiac conduction and suggesting a likely role in the favorable reprogramming of the cardiac electrophysiologic substrate after STAR.

### 25 Gy IR dynamically regulates repolarization in hiPSC-CM models.

**Figure 5:**
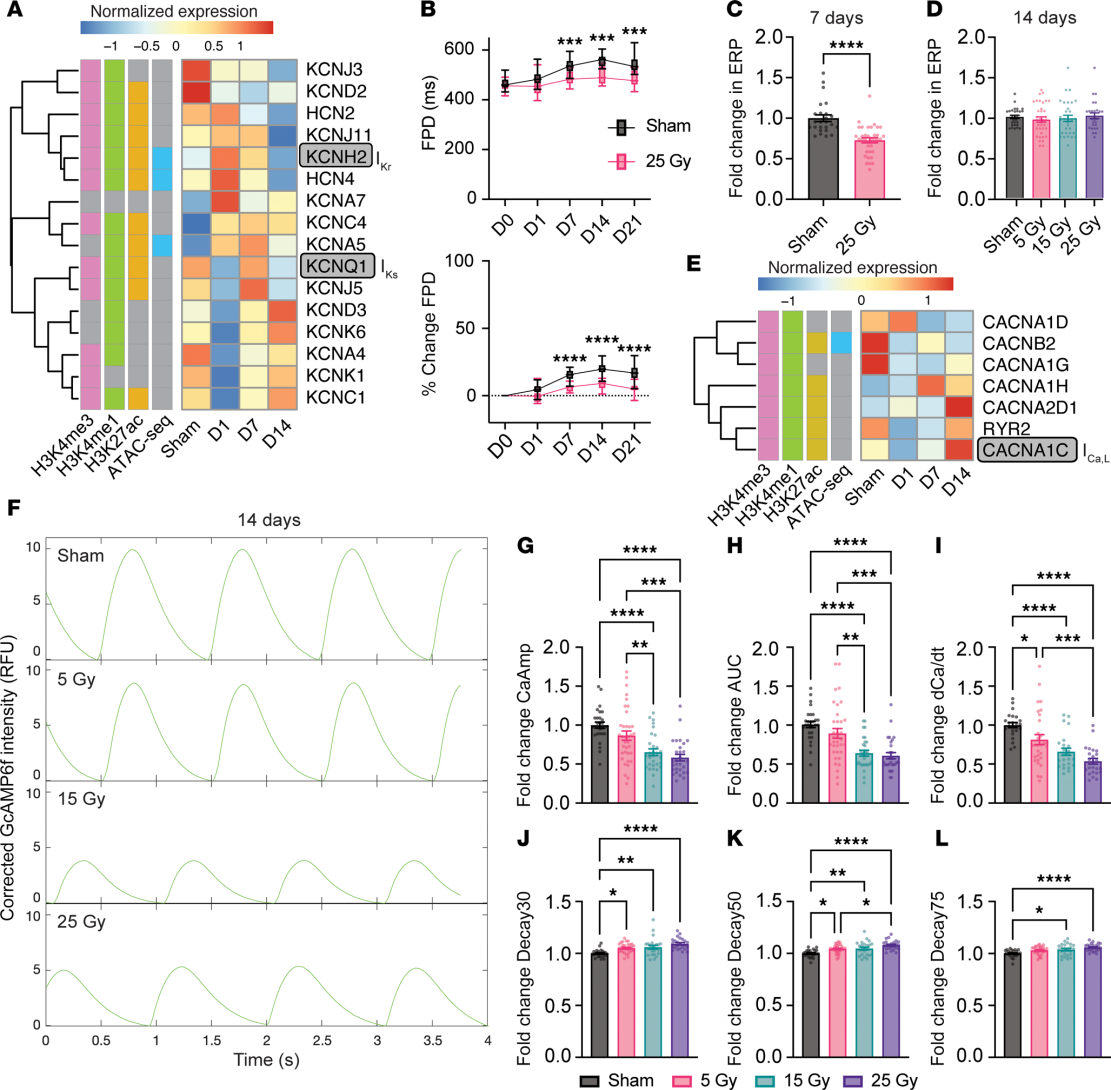
Epigenetic profiling reveals changes to repolarization and calcium handling after IR in hiPSC-CMs. (**A**) Heatmap of transcripts and epigenetic changes for potassium channels involved in the cardiac action potential. (**B**) FPD measurements assessed by MEA over time shown as FPD and the percentage change in FPD from day 0. Boxes represent 25th^_^75th percentiles, and whiskers represent minimum-to-maximum values (*n*_wells_ = 24 sham, 12 irradiated from 3 cryovials; 2-way ANOVA: *P*_time,_ *P*_treatment_, and *P*_interaction_ < 0.0001; Šídák’s post hoc test: ***P_adj_ < 0.0005, ****P_adj_ < 0.0001). (**C**) Quantified ERP from optocardiography in μEHTs 7 days after sham or 25 Gy IR, shown as the FC relative to the sham average by batch (≥25 μEHTs per condition from 3 differentiations; 2-tailed *t* test: ****P < 0.0001). (**D**) Quantified ERP from optocardiography in μEHTs 14 days after sham treatment or 5, 15, or 25 Gy IR, shown as the FC relative to the sham average by batch (≥27 μEHTs per condition from 5 independent differentiations; 1-way ANOVA: *P* = 0.76). (**E**) Heatmap of transcripts and epigenetic changes for calcium channels involved in cardiomyocyte excitation-contraction coupling. (**F**) Representative traces of relative GCaMP6 fluorescence intensity during 1 Hz pacing for tissues 2 weeks after sham treatment or 5, 15, or 25 Gy IR. (**G**–**L**) Quantification of calcium-handling parameters (CaAmp, calcium amplitude) (**G**); FC in the AUC (**H**); FC in dCa/dt (change in GCaMP fluorescence/time) (**I)**; FC in Decay30 (**J**); FC in Decay50 (**K**); and FC in Decay75 (**L**) by GCaMP6 fluorescence in sham-treated versus 5, 15, and 25 Gy irradiated μEHTs 14 days after treatment, presented as the FC relative to the sham average by batch (≥23 μEHTs from 5 independent differentiations; 1-way ANOVA: *P*_Decay75_ = 0.0002, all others < 0.0001; Tukey’s post hoc test: *P_adj_ ≤ 0.05, **P_adj_ ≤ 0.01, ***P_adj_ ≤ 0.001, ****P_adj_ ≤ 0.0001).

The ERP is another determinant of the likelihood of sustained reentry, and modulating refractoriness is an established antiarrhythmic strategy (2). We observed dynamic changes in transcription and the chromatin state of numerous potassium channels involved in cardiac repolarization (Figure 5A, Supplemental Figure 7B, Supplemental Figure 8D, and Supplemental Tables 5 and 6). Many of these channels displayed bidirectional patterns of change over time after IR, with transcript levels changing one direction early and the opposite direction late. *KCNH2* (encodes hERG, which generates the current *I*_Kr_) and *KCNQ1* (encodes KVLQT1, which generates the current *I*_Ks_) are key contributors to phase 3 of the action potential and, therefore, affect the speed of repolarization (38). Early upregulation of *KCNH2* was followed by decreased expression of both *KCNH2* and *KCNQ1* at 2 weeks (Figure 5A). There was also epigenetic remodeling around both the *KCNH2* and *KCNQ1* loci, including durably reduced chromatin accessibility at *KCNH2* (WTC cluster 2, Supplemental Figure 8D). We hypothesized that these changes would lead to faster repolarization (reduced ERP) early, followed by a prolongation of the ERP at later time points.

To test whether changes in expression and the chromatin state of potassium channel genes correlate with functional electrophysiologic reprogramming, we measured field potential duration (FPD) in iCells over time after 25 Gy IR. Both sham-treated and irradiated cells demonstrated increased FPD over time, although this increase was greater in sham-treated cells than in irradiated cells at day 21 (5.3% ± 1.1% versus 16.7% ± 1.2%; adjusted *P* value [*P*_adj_] < 0.0001; Figure 5B). We also measured the ERP in μEHTs 1 week after 25 Gy IR and observed an approximately 25% reduction in ERP in irradiated μEHTs compared with sham (*P* < 0.0001; Figure 5C). Consistent with the late downregulation of potassium channel transcripts, the ERP returned to baseline by day 14, with no significant difference detected in sham-treated tissues versus 5, 15, or 25 Gy tissues (Figure 5D). Although we did not observe ERP prolongation, as we predicted, physiologic changes often lag behind transcriptomic changes, and the relatively short timeframe of our experiments may not have captured late phenotypic changes. Still, continued reprogramming at these sites suggests that the ERP may be prolonged at late time points after IR, consistent with our recently reported findings in porcine models, in which ERP prolongation is predicted to partially underly the antiarrhythmic response to radiation (16).

### IR induces dose-dependent reductions in calcium flux in engineered heart tissue models.

Calcium is essential to cardiomyocyte function, contributing to the cardiac action potential, cardiomyocyte contraction, and intracellular signaling (38). Like potassium channels, our study showed that key calcium-handling genes displayed bidirectional patterns of change after IR, most notably early downregulation followed by upregulation of *CACNA1C* (encodes the voltage-gated calcium channel CaV1.2, which generates the current *I*_Ca,L_, contributing to phase 2 of the cardiac action potential) and *RYR2*, which facilitates calcium-mediated calcium release from the sarcoplasmic reticulum during excitation-contraction coupling (Figure 5E) (38). We also observed that less accessible DARs were enriched near genes associated with GO terms involved in cardiac contraction, such as “regulation of muscle contraction,” “sarcomere organization,” and “cardiac muscle tissue development,” which include calcium-handling genes (Supplemental Figure 7B and Supplemental Figure 8D). These findings suggest dynamic transcriptomic and epigenetic remodeling around calcium-handling genes.

We hypothesized that these epigenetic changes would correlate to acute reductions followed by late increases in calcium flux. WTC cells constitutively express the calcium-sensitive fluorescent protein GCaMP6 (39), which allows for optical measurement of calcium transients. Compared with sham tissues, tissues treated with 15 or 25 Gy displayed reduced calcium amplitude (25 Gy FC = 0.58 ± 0.04, *P*_adj_ < 0.0001; 15 Gy FC = 0.65 ± 0.04, *P*_adj_ < 0.0001), AUC (25 Gy FC = 0.61 ± 0.04, *P*_adj_ < 0.0001; 15 Gy FC = 0.64 ± 0.04, *P*_adj_ < 0.0001), and dCa/dt (25 Gy FC = 0.54 ± 0.04, *P*_adj_ < 0.0001; 15 Gy FC = 0.66 ± 0.05, *P*_adj_ < 0.0001) (Figure 5, F–I). The results for tissues treated with 5 Gy were not statistically different from those for sham-treated tissues with regard to calcium amplitude (FC = 0.87 ± 0.06, *P*_adj_ = 0.19) or AUC (FC = 0.89 ± 0.06, *P*_adj_ = 0.33), with the 5 Gy–treated tissues exhibiting a more modest reduction in dCa/dt (FC = 0.81 ± 0.06, *P*_adj_ = 0.04). To further characterize calcium handling changes after IR, we also quantified calcium decay by measuring time from peak calcium signal 70% (Decay30), 50% (Decay50), and 25% (Decay75) of that peak amplitude. There were slight but statistically significant increases in Decay30 (FC = 1.09 ± 0.01, *P*_adj_ < 0.0001), Decay50 (FC = 1.08 ± 0.01, *P*_adj_ < 0.0001), and Decay75 (FC = 1.06 ± 0.01, *P*_adj_ < 0.0001) after 25 Gy only (Figure 5, J–L). We observed similar changes 1 week after 25 Gy IR (Supplemental Figure 9, A–G).

Overall, we observed reduced calcium cycling during cardiomyocyte excitation-contraction coupling after high-dose (15 and 25 Gy), but not low-dose (5 Gy), IR. Given our transcriptomics findings, this is likely due reductions in the L-type calcium current (*I*_Ca,L_) — which would be consistent with a shortened ERP — and reduced calcium-mediated calcium release from the sarcoplasmic reticulum, which would be predicted to lead to weaker contractile function. Given the late increase in transcript levels of *CACNA1C* and *RYR2*, we predict that these trends would reverse over longer timeframes (leading to an antiarrhythmic, prolonged ERP and improving contractile function), although our relatively short time points did not capture late phenotypic changes.

### IR transcriptionally modulates cardiomyocyte mitochondrial metabolism.

**Figure 6:**
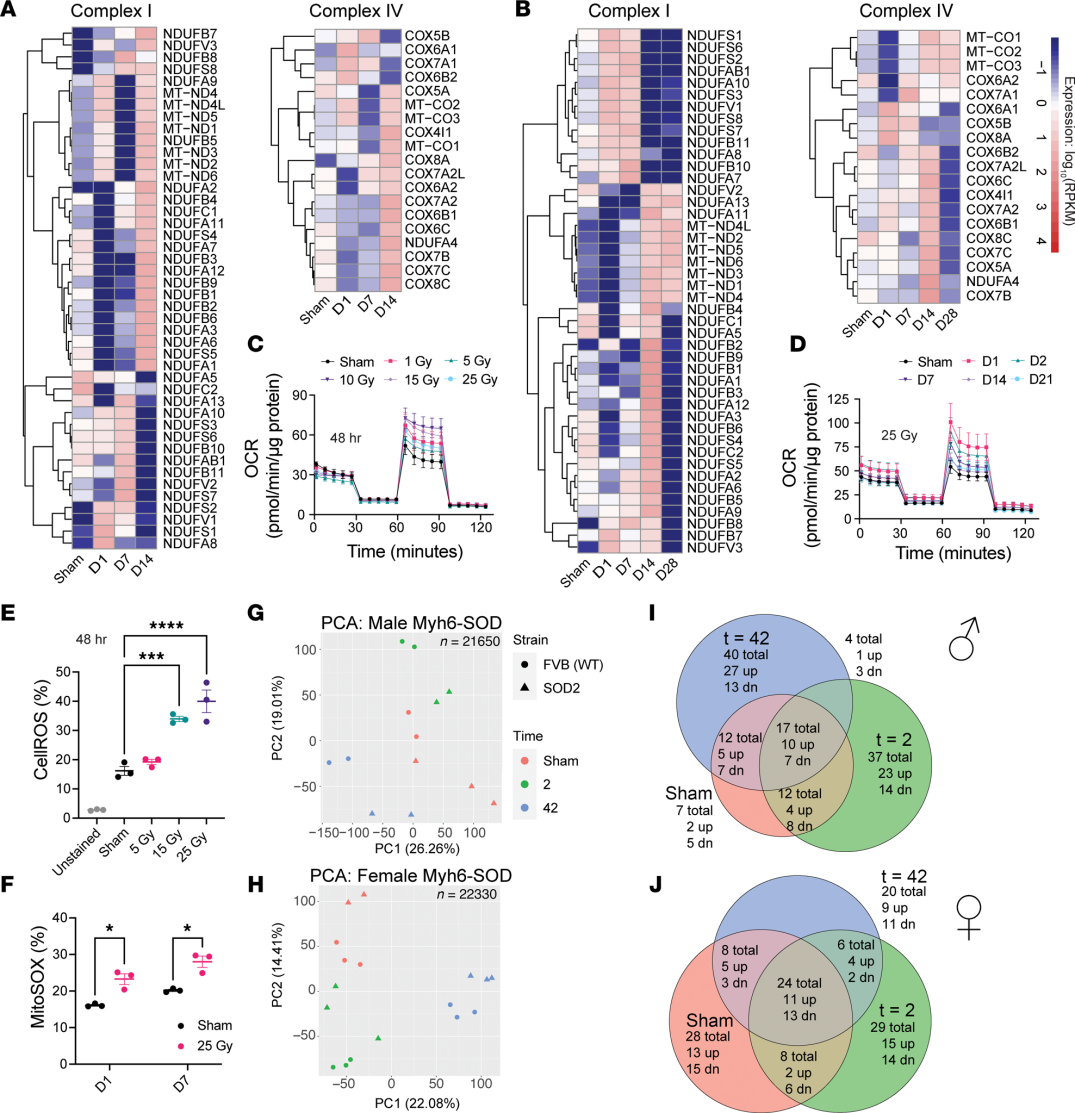
IR transcriptionally regulates mitochondrial physiology and OXPHOS. (**A** and **B**) Heatmaps for transcript levels of genes in mitochondrial respiratory chain complex I and IV for iCells (**A**) and WTC hiPSC-CMs (**B**) over time after 25 Gy IR. RPKM, reads per kilobase per million mapped reads. (**C** and **D**) OCR in iCells 48 hours after varying IR doses (**C**) or over time after 25 Gy (**D**) (*n* ≥10 replicates from 1 cryovial; OCR, oxygen consumption rate). (**E**) Percentage of cells positive for CellROS by flow cytometry 48 hours after 25 Gy in iCells (*n* = 3 replicates; 1-way ANOVA: *P* < 0.0001; Tukey’s post hoc test: ***P_adj,sham–15Gy_ = 0.0002, ****P_adj,sham–25Gy_ < 0.0001). (**F**) Percentage of cells positive for MitoSOX by flow cytometry 1 and 7 days after 25 Gy in iCells (*n* = 3 replicates; unpaired *t* tests: day 1 *P = 0.008, day 7 *P = 0.008). (**G** and **H**) PCA plots from Myh6-SOD versus WT mice after sham treatment, 2 days after IR, and 6 weeks after IR for male (**G**) and female (**H**) mice. (**I** and **J**) Euler plots showing overlap of DEGs across time points in Myh6-SOD mice after 25 Gy in male (**I**) and female (**J**) mice. Up, higher expression in WT mice; dn, lower expression in WT mice.

Calcium also plays a crucial role in mitochondrial function, including ATP production and regulation of mitochondria metabolism (40). We noted epigenetic changes at enhancers enriched for genes involved in GO terms such as “regulation of mitochondrial membrane permeability” and “regulation of calcium ion transmembrane transport” (Figure 4C). DEGs were enriched for GO terms involved in metabolism, such as “glycolytic process” (iCell cluster 1, Figure 4B), “aerobic electron transport chain,” “oxidative phosphorylation,” and “mitochondrial ATP synthesis–coupled electron transport” (WTC cluster 6, Supplemental Figure 8B). Mitochondrial OXPHOS is crucial for cardiomyocytes, given the high demand for energy (22). Examination of genes related to mitochondrial function revealed that IR upregulated genes in mitochondrial respiratory chain complexes I and IV (Figure 6, A and B). These gene expression changes were dynamic, with many increasing by day 14 and subsequently decreasing by day 28. Notably, IR increased the expression of mitochondria-encoding genes, such as *MT-ND1*, *MT-ND3*, and *MT-ND4*, at late time points in both cell lines (Figure 6, A and B).

To investigate whether IR affects the mitochondrial oxygen consumption rate (OCR) in cardiomyocytes, we conducted a Seahorse extracellular flux assay. The OCR in iCells was measured 48 hours after 1–25 Gy IR. Surprisingly, the OCR was increased at all doses after IR (Figure 6C). To determine the persistence of the effect on oxygen consumption, hiPSC-CMs were treated with 25 Gy followed by OCR assessment over time, which demonstrated acute increases that returned near baseline by day 21 (Figure 6D). These findings suggest that IR enhanced mitochondrial OXPHOS in cardiomyocytes, most likely due to the upregulation of genes associated with mitochondrial respiratory chain complexes I and IV, especially mitochondria-encoding genes, although the effect appeared transient.

### Radiation increases cellular and mitochondrial ROS in a dose-dependent fashion.

As a byproduct of increased metabolic output, increased OXPHOS leads to increased production of mitochondrial ROS (41). Radiation also produces ROS directly through radiolysis of water molecules (22). Both mitochondrial dysfunction and excess ROS are pathogenic in the heart, and tightly regulated levels of ROS are crucial signaling molecules in normal cardiomyocyte function (42). To better characterize the role of ROS in the cardiomyocyte response to IR, we quantified ROS levels in hiPSC-CMs after varying doses of IR, which revealed a dose-dependent increase in ROS 48 hours after IR (Figure 6E). We also quantified mitochondrial superoxide (MitoSOX), which was increased in irradiated cells relative to sham-treated cells at 1 and 7 days after 25 Gy (Figure 6F).

### Improved ROS scavenging via SOD2 overexpression has a modest effect on the post-IR cardiomyocyte transcriptome.

ROS can mediate changes to gene transcription (43, 44). To test whether ROS contribute to post-IR transcriptomic changes, we utilized a myosin heavy chain 6-superoxide dismutase 2 (Myh6-SOD) mouse, which overexpresses SOD2 — an enzyme that converts superoxide to hydrogen peroxide — in cardiomyocytes, resulting in enhanced ROS scavenging (45). RNA-seq demonstrated distinct transcriptional states of Myh6-SOD mice versus WT littermates in both sexes at baseline (sham), which persisted 2 days and 6 weeks after 25 Gy whole-heart IR; however, the number of DEGs was surprisingly small (Figure 6, G–J). Many of these DEGs were present in the sham condition and not unique to post-IR states, suggesting they were not drivers of post-IR physiology, but rather baseline differences between Myh6-SOD and WT mice. Small numbers of DEGs were unique to the day-2 or week-6 post-IR condition, and very few DEGs were shared across post-IR time points only (Figure 6, I and J).

Some interesting themes do emerge from these DEGs (Supplemental Table 15). For example, mitochondrial complex I and mitochondrial tRNAs were downregulated in WT mice relative to Myh6-SOD mice after IR, suggesting that ROS reduce the expression of mitochondrial genes. Some sarcomeric genes (*Myh7*, *Capn3*) also appear downregulated by ROS. Genes involved in responding to cellular stress, such as the hypoxia-responsive gene *Egln3* and the p53 target *Phlda1* (46), were activated by ROS. Although interesting, the small number of genes suggests a modest role for superoxide in regulating gene expression changes after IR.

### Unbiased cross-species comparison identifies p53 as a candidate regulator of the cardiomyocyte response to IR.

**Figure 7:**
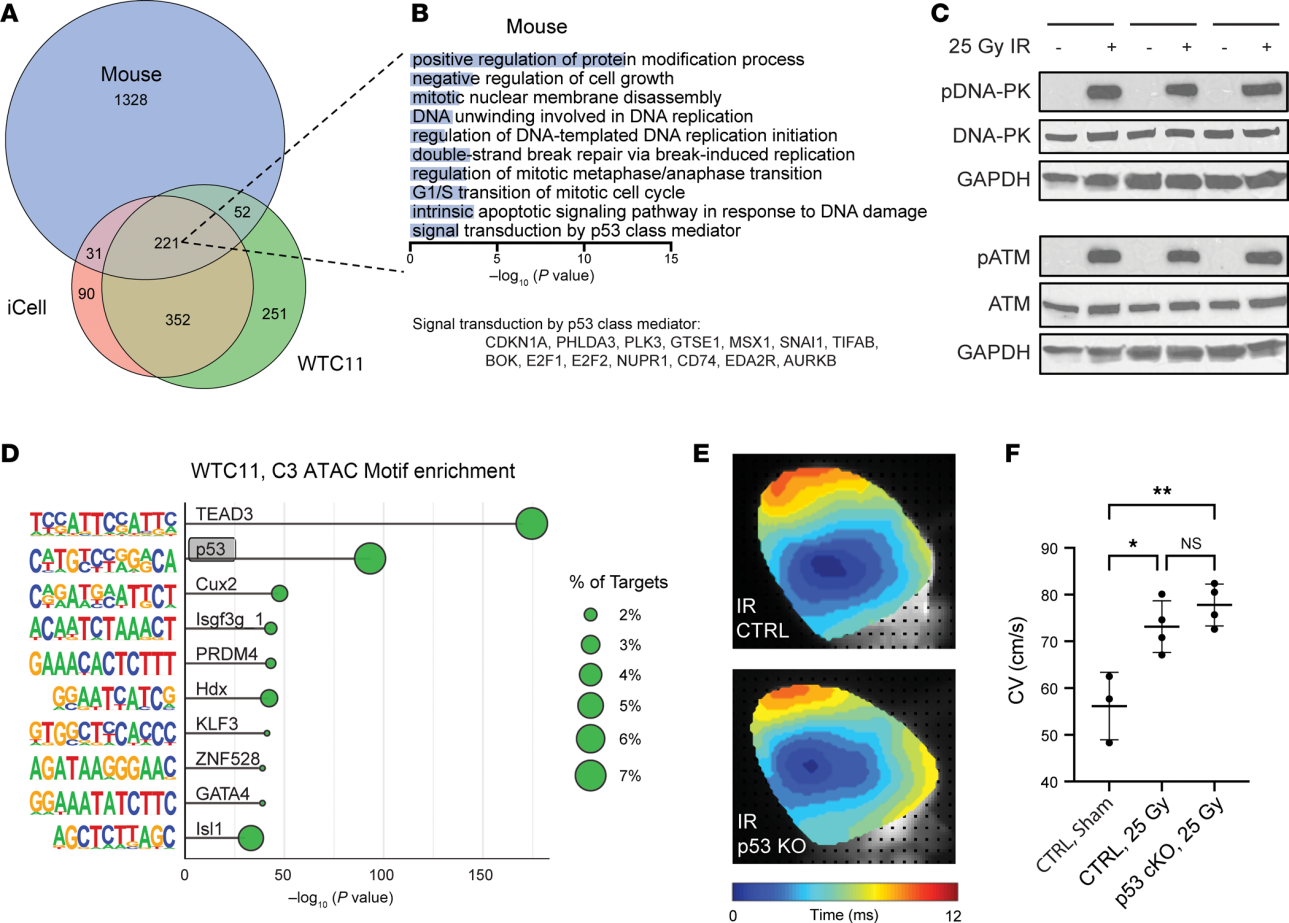
Cross-species comparison demonstrates conserved transcriptional pathways in mice and humans after IR. (**A**) Euler diagram showing overlap of GO pathways enriched at any time point among DEGs across model systems. (**B**) Selected representative pathways shared across murine and both hiPSC-CM cell lines. *P* values shown are from murine data. Genes from the GO term “signal transduction by p53 class mediator,” which were differentially expressed in mice, are highlighted below. (**C**) Western blots probed for ATM, phosphorylated ATM (p-ATM), DNA-PK, p–DNA-PK, and GAPDH in sham-treated versus 25 Gy irradiated WTC hiPSC-CMs 30 minutes after IR (*n* = 3 differentiations). (**D**) HOMER Motif analysis of TF binding motifs enriched in DARs in WTC cluster 3. (**E**) Representative activation maps for p53-cKO (αMHC-MerCreMer;p53^fl/fl^ plus tamoxifen) and control (CTRL) (αMHC-MerCreMer;p53^WT/WT^ plus tamoxifen) mice 6 weeks after 25 Gy whole-heart IR. (**F**) Quantified CV (±SD) in p53-cKO and control mice 6 weeks after sham treatment or 25 Gy IR (1-way ANOVA: *P* = 0.0028; Tukey’s post hoc test: *P_adj,ctrl+sham-ctrl+25Gy_ = 0.011, **P_adj,ctrl+sham-cKO+25Gy_ = 0.0027, *P*_adj,ctrl+25Gy-cKO+25Gy_ = 0.51).

To identify additional potential mediators of cardiomyocyte electrical reprogramming after IR, we conducted an unbiased comparison across our hiPSC-CM and murine RNA-seq datasets at the pathway level. Because of the complexity of comparing variable time courses across multiple models, pathways enriched in each model at any time were compared. Most differentially regulated pathways identified in iCells were also identified in WTC hiPSC-CMs (82%), and vice versa (65%; Figure 7A). Unsurprisingly, many pathways were unique to either mice or hiPSC-CMs; however, 221 pathways were enriched at some time across all 3 models, and 30% of pathways enriched in 1 or more hiPSC-CM line were also identified in murine cardiomyocyte nuclei, despite differences in time points, species, and cellular maturity (Figure 7A and Supplemental Table 16). Most notably, this overlap included pathways involved in apoptosis, cell-cycle arrest, and the DDR (Figure 7B). To further probe the role of the DDR, we performed Western blotting for phosphorylated (activated) ataxia telangiectasia mutated (ATM) and DNA-dependent protein kinase (DNA-PK), 2 core kinases involved in responding to DNA double-stranded breaks (DSBs) (47). This revealed robust activation of both ATM and DNA-PK in hiPSC-CMs 30 minutes after 25 Gy IR (Figure 7C). Across sequencing modalities, we noted enrichment of p53-related pathways (Figure 7B), which are downstream of ATM and DNA-PK activation (47). For example, we observed enrichment of the p53 pathway among genes acutely upregulated by IR in hiPSC-CMS, with corresponding enrichment of cell-cycle genes in downregulated DEGs (Figure 4B). Additionally, the p53 binding motif was enriched in acutely opening DARs after IR in WTC hiPSC-CMs (Figure 7D), as well as in regions with increased H3K4me1 and H3K4me3 (Figure 4E). These data demonstrate activation of canonical DDRs to DSBs that could then regulate the post-IR transcriptional state.

### Loss of cardiomyocyte p53 does not affect radiation-induced conduction reprogramming.

p53 is activated in response to cellular stressors, including DNA damage, and activated p53 functions as a TF to broadly regulate gene expression and control cellular processes such as DNA damage repair, cell-cycle arrest, senescence, and apoptosis (48). Given this prominent role of p53 in responding to cellular stressors such as DNA damage, as well as p53’s role as a potent TF in the heart (49), we hypothesized that p53 activation may mediate electrophysiologic reprogramming after IR through transcriptional activation of the sodium channel *Scn5a*. To test this hypothesis, we generated a tamoxifen-inducible, cardiomyocyte-specific p53-KO mouse (p53-cKO) using a Cre recombinase under the cardiomyocyte-specific myosin heavy chain promoter with loxP-flanked *Trp53*. Adult mice underwent tamoxifen-induced Cre recombination and washout. Optical mapping was performed 6 weeks after 25 Gy whole-heart IR or sham treatment. Irradiated littermates receiving tamoxifen and expressing Cre recombinase without floxed *Trp53* were used as controls. Despite p53’s crucial role in responding to DNA damage, none of the p53-cKO mice expired after IR, and histology demonstrated an overall normal structure without increased fibrosis (Supplemental Figure 10, A and B). Both WT and p53-cKO mice showed increased CV compared with nonirradiated controls; however, p53-cKO mice showed no difference in CV compared with irradiated control mice expressing p53 (Figure 7, E and F). These data demonstrate that cardiomyocyte-specific p53 was not necessary to increase CV after 25 Gy IR, suggesting that other redundant pathways may be involved in sensing IR-induced DNA damage in cardiomyocytes to mediate electrophysiologic reprogramming.

### Cross-species comparison identifies potential transcriptional regulators of the cardiomyocyte response to IR.

We next asked whether TF binding motifs enriched in murine DARs were shared in the hiPSC-CM models. We found that 3 TFs had binding motifs enriched in all 3 experimental models: GATA2, Tbx20, and NFIC. We also noted several families of TFs that were enriched across models (Supplemental Figure 11). For example, in addition to GATA2, several other GATA family members displayed binding motif enrichment, including GATA1 and the cardiac TFs GATA4 and GATA5 (GATA4/-5). Unlike GATA4 and GATA5, GATA1 and GATA2 are not highly expressed in cardiac tissue; however, GATA family TFs share substantial homology of DNA binding domains (50), so the GATA1/-2 motifs identified by our enrichment analysis may represent regions bound by GATA4/-5. Similarly, we noted enrichment of several MEF2 family members (MEF2a/b/d). Although MEF2c is best recognized in the heart, the binding sequence of MEF2 TFs is highly conserved with similar binding motifs, much like in the GATA family (51). All models also had enrichment of transcriptional enhanced associate domain (TEAD) family binding sites in at least 1 DAR cluster, which are required for cardiogenesis and are crucial negative regulators of cellular proliferation through the Hippo pathway (52). This analysis identified additional candidate TFs that may be involved in regulating the cardiomyocyte response to IR.

## Discussion

STAR is an important emerging modality for treating refractory VT; however, the mechanism(s) behind its therapeutic effect remains unclear. Growing evidence suggests that fibrosis and tissue destruction cannot account for STAR’s antiarrhythmic effect (6, 7, 15). Instead, STAR favorably alters the electrical substrate via persistent changes in ion channel expression (15, 18–20). In the present study, we explored how single-fraction IR induces durable changes in ion channel expression and electrophysiology. Our findings implicate chromatin dynamics and epigenetic memory in the electrophysiologic reprogramming that underlies the therapeutic response to STAR and provide insight into other post-IR changes in cardiomyocyte physiology.

Little is known about the cardiomyocyte response to radiation, as many of the previous studies were performed in mitotically active cells, such as cancer cells. Existing data regarding cardiac radiation focus on occupational or environmental exposures (e.g., nuclear disasters or space travel) and on thoracic cancer radiotherapy to which the heart was unavoidably exposed. These observations are challenging to generalize to patients who undergo STAR, given the differences in dose and fractionation, radiation modality, and patient populations (22). Characterizing the molecular and physiologic changes in cardiomyocytes after high-dose, single-fraction IR is therefore crucial to understanding the therapeutic (and potentially toxic) effects of STAR.

We found durable changes to nuclear morphology in irradiated human and murine myocardium, implicating intranuclear processes. Epigenetic factors are key regulators of nucleus size and shape due to physical interactions between chromatin and lamin (27). Chromatin-lamin interactions also regulate gene expression during cardiac differentiation and lineage restriction (53). We therefore speculated that these changes to nuclear morphology may be indirect evidence of chromatin remodeling.

Our multimodal sequencing approach revealed that post-IR cardiomyocytes had distinct transcriptomic and epigenomic profiles within 24–48 hours of IR in both mice and hiPSC-CMs. More interestingly, although many of the acute changes resolved, a subset persisted out to 6 weeks after IR in mice and 4 weeks after IR in hiPSC-CMs. Dynamic changes to chromatin accessibility and epigenetic remodeling of enhancer regions were observed in genomic regions enriched for genes involved in cardiac depolarization and conduction, most notably persistent increases in accessibility at the TSS (mice) and in epigenetic markers of active transcription (iCells) at the *SCN5A* locus. These findings suggest a role for chromatin dynamics and enhancer-mediated regulation in the beneficial electrophysiologic reprogramming after IR and support the presence of epigenetic memory after IR in cardiomyocytes, which could explain durable reductions in VT in the absence of scar formation.

Using multiple in vitro hiPSC-CM models, we further demonstrate that IR-mediated electrophysiologic reprogramming — including upregulation of Na_V_1.5 and increased CV — is at least partially cardiomyocyte autonomous, suggesting a role for cell-intrinsic mechanism(s), rather than cell-to-cell signaling or bystander responses. Others have also observed increased CV and ion channel upregulation in isolated cardiomyocytes, such as hiPSC-CMs and neonatal rat ventricular cardiomyocytes (NRVCs), within 1–7 days after IR (19, 20), which may help to explain the rapid onset of clinical responses. Our study, by looking at time points as late as 3 weeks in vitro, demonstrates persistent responses in an isolated cell culture system. This is a crucial finding, as a complete understanding of STAR must explain the persistent electrophysiologic and ion channel expression changes in animal models and the durability of the clinical response.

In our study, IR appeared to regulate multiple aspects of cardiomyocyte biology. Specifically, numerous cardiac-relevant potassium and calcium channels demonstrated fluctuating expression with dynamic chromatin remodeling at nearby genomic regions. Calcium and potassium currents are known to determine the cardiomyocyte refractory period: increased potassium currents accelerate repolarization, and increased calcium flux leads to a longer plateau phase that prolongs repolarization (38). Because the capacity for a circuit to sustain reentry is dependent on the mathematical product of CV and the ERP, an increasing ERP is antiarrhythmic (2). Border zone myocardium exhibits particularly heterogeneous action potential durations, increasing vulnerability to reentrant arrhythmias (54). Our group recently reported increased refractoriness after STAR in a porcine model of myocardial infarction, representing an additional mechanism beyond increased CV by which STAR may be used to treat VT (16). Dynamic epigenetic modulation of calcium and potassium channel genes may contribute to prolonging and homogenizing the ERP to reduce vulnerability to reentry. Phenotypically, the refractory period of hiPSC-CMs was shortened acutely after IR, consistent with recently published findings in NRVCs (19). In our studies, the ERP normalized relative to sham by 2 weeks after IR, with corresponding late downregulation of potassium channels and upregulation of calcium channels, suggesting a transient effect. Given our recent porcine findings, we speculate that this dynamic remodeling may continue and prolong the ERP over time.

Downregulation of calcium channels (especially the L-type calcium channel: *CACNA1C*; Ca_V_1.2) with corresponding reductions in calcium flux would also be expected to impair excitation-contraction coupling, weakening contractility. This contrasts with published findings, which show no reductions in left ventricular ejection fraction (LVEF) in patients after STAR (6, 7, 12, 55) and even suggest improved ventricular function (56). It is also contrary to reports of increased calcium flux at 96 hours after 20 Gy in NRVCs (19) and upregulation of Ryr2 and Ca_v_1 at 7 days after 25 Gy in rat hearts (57). As with potassium channels, we observed a late upregulation of *CACNA1C*, which we predict would lead to normalization or even increases in calcium flux over a longer experimental period.

Studies reported in current literature demonstrate mitochondrial dysfunction after IR in mice, in vitro models, and in human cohort studies (22, 58–60); however, these studies focused on low doses delivered in a fractionated or continuous fashion, as opposed to single-fraction, high-dose IR as implemented with STAR. We therefore know very little about the metabolic response of cardiomyocytes after STAR. We found that mitochondrial respiratory chain complexes I and IV were temporarily upregulated after IR. Consistent with these gene expression changes, but in contrast to previous literature reporting other radiation dosing schedules, we found that the OCR was acutely increased at all doses tested, with levels returning to just above baseline by day 21. This finding demonstrates differential metabolic responses based on the dosing strategy and suggests that STAR may transiently alter mitochondrial metabolism in ways that are beneficial for contractility.

Both increased mitochondrial OXPHOS and direct effects of IR are expected to increase production of ROS (22, 41). Indeed, 25 Gy increased cellular and mitochondrial ROS; however, the increases were modest, suggesting that cardiomyocytes compensate through ROS scavenging. ROS can modulate gene expression by triggering recruitment of transcriptional and epigenetic regulatory machinery as part of the base excision repair pathway responding to DNA damage (43, 44); however, improved ROS scavenging via overexpression of SOD2 in Myh6-SOD mice revealed only a small number of DEGs after IR, suggesting a modest role for ROS in regulating post-STAR gene expression.

We also explored differential effects based on IR dose. Twenty-five Gy was initially chosen for clinical studies as a minimal dose that might induce fibrosis while also minimizing toxicity; however, as our understanding of STAR’s therapeutic mechanism evolves, optimizing the dose to maximize benefit is of great clinical importance. There is evidence that lower doses may be effective. Preclinical studies with mice and NRVCs demonstrated increased CV and ion channel expression at doses of 15 and 20 Gy, respectively (15, 19), and 1 case study reported a clinical response for a patient treated with 12 Gy (61). Furthermore, although 25 Gy ore more is delivered to the PTV, surrounding regions of the heart receive a range of doses. Understanding cardiomyocyte responses to the full range of clinically received doses is therefore crucial. We observed no change in CV in μEHTs after 5 Gy, a small, nonsignificant increase after 15 Gy, and a larger, statistically significant increase in CV after 25 Gy. The disparity in response at 15 Gy between the present study and our prior murine data may again suggest that cardiomyocyte cell-autonomous mechanisms explain only part of the CV increases, and that maximal reprogramming efficiency relies on a multifactorial mechanism involving cell-to-cell interactions and the cellular microenvironment. Alternatively, it may be the result of hiPSC-CM immaturity or differing time points of CV measurement after IR across studies.

Although STAR has a favorable safety profile, up to 15% of patients experience major adverse events attributable to the procedure (21). Minimizing potential toxicities while achieving the desired therapeutic effect is therefore another goal of dose titration. Toward this end, we found that calcium flux was reduced at 15 Gy and 25 Gy, but only minimally so after 5 Gy. Interestingly, 5 Gy is the mean dose to which the heart is exposed in patients prescribed 25 Gy for STAR, and whole-heart IR has been shown to preserve left ventricular function and attenuate adverse remodeling in murine cardiac injury models (56). Our dose-response data suggest that there may be optimal dosing strategies to minimize harmful responses (like reduced calcium flux) to nontargeted regions of the heart while maintaining beneficial antiarrhythmic effects in targeted myocardium.

Last, we explored additional potential mechanisms of cellular reprogramming. The p53 pathway was activated after IR, consistent with its canonical role in responding to DNA damage (48). In the heart, p53 regulates mitochondrial biogenesis, glucose metabolism, and the response to pressure overload (49). p53 also regulates the epigenome through interaction with epigenetic machinery (62). We generated cardiomyocyte-specific p53-KO mice to test whether p53 mediates post-IR electrophysiologic reprogramming. IR in mice lacking p53 in cardiac endothelial cells results in vascular loss, myocardial necrosis, and heart failure (63). In contrast, our cardiomyocyte-specific p53-cKO mice remained viable, demonstrated grossly normal cardiac histology, and retained accelerated CV after IR. We conclude that p53 is not necessary for CV reprogramming and that alternate pathways exist for responding to damage after IR in cardiomyocytes, although p53 may regulate other post-IR changes in cardiomyocytes.

Using motif enrichment analysis, we identified additional TFs that may play a role in cardiomyocyte responses to IR, including several that were shared across model systems: Tbx20 and the GATA, MEF2, and TEAD families. GATA TFs are involved in cardiac development and disease, particularly GATA4, which plays a key role in regulating *Scn5a* transcription (50, 64). MEF2 family binding sites were also enriched in both mouse DARs and DMRs for H3K27ac and H3K4me1. MEF2 TFs (especially MEF2c) interact with GATA TFs in the heart and regulate cardiomyocyte differentiation, cardiac conduction, metabolism, and stress responses such as hypertrophy and fibrosis (51, 65). Tbx20 also broadly regulates the transcription of cardiac genes (including *Scn5a*) in part via recruitment of other cardiac TFs including MEF2C, GATA4, and Tbx5 (66). Both GATA4 and Tbx20 motifs are present in known superenhancer regions that regulate *Scn5a* expression (30). This analysis identifies several biologically plausible regulators of the cardiomyocyte response to IR, many of which are known to interact as part of a transcriptional regulatory network and which warrant further investigation in future studies.

Given that the detailed mechanistic link between IR and electrophysiologically meaningful epigenetic reprogramming of cardiomyocytes remains elusive, we speculate that DDR pathways may play an important role. Interestingly, the cellular response to DNA damage involves the chromatin remodeling required for downstream DDR signaling (23, 24). Multiple chromatin remodelers are recruited to DSBs as part of the DDR (23, 24). The long-term effects of this chromatin remodeling remain unclear. During the DDR, early changes in the chromatin state allow for successful DNA repair prior to “restoring” chromatin organization; however, this chromatin restoration may not faithfully reproduce the initial configuration but instead may leave a “damage imprint” (23). Other cell types display epigenetic memory after radiation. For example, in fibroblasts, epigenetic memory has been implicated in impaired wound healing, a lasting adverse effect after radiation (67). This notion is further supported by a recent study demonstrating that after successful DSB repair, alterations to chromatin architecture persist and affect the transcription of nearby genes (68). Durable modifications to the epigenetic landscape could explain the persistent changes in gene expression and electrophysiologic phenotype after a single dose of radiation in our cardiomyocyte studies, representing true cellular reprogramming. Future work to identify clear effectors linking IR to the formation of this “damage imprint” in cardiomyocytes will be of great importance.

A limitation of this work is the immaturity of hiPSC-CMs, which more closely resemble neonatal cardiomyocytes than adult myocardium (32). hiPSC-CMs display spontaneous beating, slow CV, a lack of some potassium currents (e.g., *I*_K1_), underdeveloped calcium-handling machinery, and a reliance on glycolysis over fatty acid metabolism (69). hiPSC-CMs also retain some proliferative capacity, which likely makes them more radiosensitive than adult cardiomyocytes (70). Furthermore, the difficulties of prolonged culturing for some in vitro models limited our ability to study longer-term responses. To mitigate these limitations, we matured hiPSC-CMs utilizing μEHTs, and we validated the key findings and explored late time points in mice. Another limitation is that our murine data were generated from healthy mice rather than diseased ones, although we previously demonstrated Na_V_1.5 and Cx43 upregulation in infarcted mouse hearts. Additional studies in injury models, which more accurately represent the substrate of patients receiving STAR, are needed to continue to expand our understanding of cardiac radiation. Finally, although we implicate durable changes to the epigenetic landscape in the cardiomyocyte response to IR, a complete understanding of molecular mediators of these changes remains elusive and will be an important area of future investigation.

Many questions about STAR remain, such as the optimal dose, the targeting strategy, and the timing of radiation delivery to maximize the benefit and minimize toxicity. A clearer understanding of the mechanistic basis for STAR will begin to provide insight into these questions. Toward that end, we define a role for cardiomyocyte-autonomous, temporally dynamic changes to the chromatin landscape with corresponding changes to cardiomyocyte physiology, including dose-dependent beneficial reprogramming of cardiac conduction, as well as changes to cardiac repolarization, calcium handling, and metabolism.

## Methods

### Sex as a biological variable.

Both male and female mice were used in the histology, flow cytometry, optocardiography, and RNA-seq studies. We observed baseline differences between the transcriptome of unirradiated male and female mice; however, changes after IR treatment were similar regardless of sex. Subsequent ATAC-seq experiments examined male mice only to reduce variability in unirradiated animals.

Our study utilized hiPSC-CM lines from male and female individuals. Key findings were validated across cell lines.

Additional details on the methods used in this study can be found in the Supplemental Methods.

### Statistics.

Statistical analysis and data plotting were performed using GraphPad Prism (version 10.4.1; GraphPad Software) or R environment (version 4.2.2). Unless otherwise specified, a *P* value of less than 0.05 (95% confidence) was considered statistically significant. When comparing 2 experimental groups, a 2-tailed, unpaired *t* test was used. A Wilcoxon ranked-sum test was used for comparing individual gene mRNA transcript levels. When comparing 3 or more experimental groups, a 1-way ANOVA was used with Tukey’s post hoc test if comparing all conditions or Dunnett’s post hoc test if comparing all conditions with a control. When comparing experimental groups across multiple variables, a 2-way ANOVA with Šídák’s post hoc test was used. Data are represented as the mean ± SEM.

### Study approval.

Human sample collection was approved by the Washington University School of Medicine IRB (IRB no. 201606081). Informed consent was provided prior to specimen collection. Mouse protocols were approved by the Animal Studies Committee at Washington University School of Medicine. All animals were handled in accordance with the NIH’s *Guide for the Care and Use of Laboratory Animals* (National Academies Press, Eighth edition, 2011.

### Data availability.

RNA-seq, ATAC-seq, and CUT&Tag data have been deposited in the NDBI Gene Expression Omnibus (GEO) and are available under accession numbers GSE291280 (RNA-seq), GSE291268 (ATAC-seq), and GSE291264 (CUT&Tag). All other data are available within the manuscript and in the Supporting Data Values file.

## Author contributions

SDJ and SLR primarily conceptualized the project aims and experimental design, with input from AF, SN, DMZ, JS, JKS, and BZ. SDJ, AF, DH, SN, DMZ, SM, N Hu, YX, AB, JT, NG, and SH contributed to experimental data collection. GDH provided clinical radiation treatment plans. Bioinformatics analysis was performed primarily by SF and BZ, with additional work by JS. LS provided technical support with IR. Stem cell differentiation was performed by LBB and KCW. Materials for engineered heart tissues were supplied by LA and N Huebsch. JKS, BZ, and SLR contributed funding. SDJ wrote the initial draft of the manuscript, with contributions from DH, SF, BZ, LBB, SN, and DMZ. All authors reviewed and provided input on editing the manuscript.

## Funding support

This work is the result of NIH funding, in whole or in part, and is subject to the NIH Public Access Policy. Through acceptance of this federal funding, the NIH has been given a right to make the work publicly available in PubMed Central.

- NIH grants R01 HL 163274 (to SLR and JKS) and R35GM142917 (to BZ).
- Additional Ventures Cures Collaborative (to SLR).
- Sarnoff Foundation (to SDJ and AF).
- Career Award for Medical Scientists from the Burroughs Wellcome Fund (to SLR).

## Supplementary Material

Supplemental data

Unedited blot and gel images

Supplemental table 1

Supplemental table 2

Supplemental table 3

Supplemental table 4

Supplemental table 5

Supplemental table 6

Supplemental table 7

Supplemental table 8

Supplemental table 9

Supplemental table 10

Supplemental table 11

Supplemental table 12

Supplemental table 13

Supplemental table 14

Supplemental table 15

Supplemental table 16

Supporting data values

## Version 1 02/17/2026

In-Press Preview

## Version 2 04/01/2026

Electronic publication
