# Supplementary material for: Cardiac radiotherapy–induced epigenetic memory underlies electrophysiologic and metabolic reprogramming
Source: J Clin Invest. 2026 Feb 17;136(7):e193087. doi: 10.1172/JCI193087 (PMC13038212; doi:10.1172/JCI193087)
Supplement: Supplemental data [file jci-136-193087-s008.pdf]

Supplemental Tables

Supplemental Table 1: Significant DEGs in PCM1+ cardiomyocyte nuclei from day-42 irradiated versus sham mouse ventricles.

Supplemental Table 2: GO term enrichment for DEGs from day-42 irradiated versus sham mouse ventricles.

Supplemental Table 3: Significant DARs in PCM1+ cardiomyocyte nuclei from day-2 irradiated and day-42 irradiated versus sham mouse ventricles.

Supplemental Table 4: GO term enrichment by k-means cluster for DARs from PCM1+ nuclei from mouse ventricles over time after IR.

Supplemental Table 5: Significant DEGs in day-1, day-7, and day-14 irradiated versus sham iCells.

Supplemental Table 6: Significant DEGs in day-1, day-7, day-14, and day-28 irradiated versus sham WTC hiPSC-CMs.

Supplemental Table 7: GO term enrichment for DEGs by k-means cluster in iCells over time after IR.

Supplemental Table 8: GO term enrichment for DEGs by k-means cluster in WTC hiPSC-CMs over time after IR.

Supplemental Table 9: Significant DARs in day-1, day-7, and day-14 irradiated versus sham iCells.

Supplemental Table 10: Significant DARs in day-1, day-7, day-14, and day-28 irradiated versus sham WTC hiPSC-CMs.

Supplemental Table 11: Significantly enriched GO Biological Process terms from k-means clustered DARs iCells overtime after IR.

Supplemental Table 12: Significantly enriched GO Biological Process terms from k-means clustered DARs WTC hiPSC-CMs overtime after IR.

Supplemental Table 13: DMRs for H3K27ac, H3K4me1, H3K4me3, and H3K27me3 CUT&Tag in iCells.

Supplemental Table 14: GO term enrichment in DMRs for H3K27ac, H3K4me1, and H3K4me3 CUT&Tag in iCells.

Supplemental Table 15: Significant DEGs in WT FVB vs Myh6-SOD mice by sex and timepoint.

Supplemental Table 16: Transcriptionally enriched GO terms shared across murine cardiomyocytes, iCells, and WTC hiPSC-CMs

Supplemental Methods

Human Specimen Collection

At the time of heart transplant or death, whole-heart specimens were obtained as previously described from one patient who had received STAR, as well as from non-failing donors (15). Targeted and remote sections were determined through comparison with the corresponding radiation treatment plan, followed by resection of these areas and fixation in 10% formalin for histopathology.

Mouse Studies

All animal studies were performed with outbred WT CD-1 mice (Charles River Laboratories, Wilmington, MA) except in experiments utilizing Myh6-SOD mice (FVB-Tg(Myh6-SOD2,Tyr)3Pne/J, Jackson Laboratory #009438) (45) or p53 cKO mice ($p53^{LoxP}$, Jackson Laboratory #008462 crossed with ɑMHC-MerCreMer, Jackson Laboratory #005657) (49, 71, 72). Mice were housed in accordance with animal study guidelines at Washington University. They were maintained in 12-hour light/dark cycles, ambient temperatures of 20-22 degrees Celsius, at a humidity range between 40-60%, with access to ad libitum food and water. Treatment was performed at 8 weeks of age. In p53 cKO experiments, mice were fed tamoxifen chow for a total of 10 days for tamoxifen-induced Cre recombination, followed by a 4-week washout prior to radiation or sham treatment. ɑMHC-MerCreMer that received tamoxifen but without floxed *Trp53* were used as controls.

Cell Culture

Two cell lines were used for hiPSC-CM experiments: iCell Cardiomyocytes^2 (FujiFilm, R1220) and WTC11-AAV-CAG-GCaMP6 (WTC). iCell Cardiomyocytes^2 (iCells) are a commercially available hiPSC-CM line derived from a healthy female donor. Cryopreserved iCells were cultured according to the manufacturer's instructions. Briefly, tissue culture plates were precoated with 0.1% gelatin in water (STEMCELL Technologies, 07903). Cells were thawed and plated in iCell Cardiomyocyte Plating Medium (FujiFilm) at the recommended density of 1.56×10^5 cells/cm^2 and subsequently cultured at 37°C and 5% CO_2. The next day, media was changed to iCell Maintenance Medium (FujiFilm), with media subsequently changed every 2-3 days. Cells were then cultured for 5-7 days to form a confluent monolayer, and synchronous spontaneous beating was confirmed before downstream experiments.

The WTC line (Coriell Repository #GM2525) is a human iPSC line from a male donor modified from the parent WTC line using genome editing to insert a single copy of CAG-driven GCaMP6f into the AAVS1 "safe harbor" locus (35) and was generously provided by Dr. Bruce Conklin (Gladstone Institute of Cardiovascular Research). iPSCs were maintained in Essential 8 Medium (E8) (ThermoFisher, A1517001). Plates were coated with human embryonic stem cell-Qualified Matrigel (Corning, 354277) diluted in DMEM/F-12 (ThermoFisher, 11330057). Cells were passaged every 4 days using Accutase (Stem

Cell Technologies, 07922) and replated at a density of 10,500 cells/cm^2 in E8 with 10 μM Y-27632 for ROCK inhibition (Tocris, 1254).

Cardiomyocytes were generated from the WTC line using small molecule modulation of Wnt signaling, as previously described (73). On differentiation day -3, iPSCs were lifted using Accutase and plated in E8 with 10 μM Y-27632 at a density of 25,000 cells/cm^2. Media was then changed daily with fresh E8. On differentiation day 0, media was changed to RPMI 1640 (ThermoFisher, 11875119), 2% B-27 minus insulin (ThermoFisher, A1895601), and 150 μg/ml L-ascorbic acid (Sigma, 4544) (R-) supplemented with 6 μM CHIR 99021 (Tocris, 4423) for 48 hours, after which media was changed to R- supplemented with 5 μM XAV939 (Tocris, 3748) and left for an additional 48 hours. At this time media was replaced with un-supplemented R-. On day 6, media was changed to RPMI 1640 with 2% B-27 Supplement (ThermoFisher, 17504044) and 1% Antibiotic-Antimycotic Solution (Corning, 30-004-CI) (R+), and cells were maintained with media changes every 2 days. Beating of the monolayer was confirmed by day 10.

Lactate-mediated metabolic selection was performed from day 10 to day 18 by culturing cells in RPMI 1640 without glucose (ThermoFisher, 11879020) plus 4 mM Sodium DL-lactate solution (Sigma, L4263), 25 mM HEPES (Corning, 25-060-CI), and 1% Antibiotic-Antimycotic (74). On differentiation day 18, media was replaced with R+ for recovery. On day 21, the cells were digested through overnight incubation at 37°C in 0.6 mg/ml Collagenase II (Worthington, LS004176) in Homemade Hank's Balanced Salt Solution (136 mM NaCl, 4.16 mM $NaHCO_3$, 0.34 mM NaH_2PO_4, 5.36 mM KCl, 0.44 KH_2PO_4, 5.55

mM dextrose, 5 mM HEPES in sterile deionized H_2O). The digestion was stopped by dilution with 2x volume of DMEM/F-12, 1 mg/ml BSA, 10 µg/ml DNase I (Sigma, 260913-10MU) (Wash Buffer). The cell suspension was centrifuged at 300 g for 3 minutes at room temperature, washed with Wash Buffer, and then centrifuged again. Cells were resuspended in R+ supplemented with 10 µM Y-27632 and plated at a density of 205,000-250,000 cells/cm^2. The next day, cells were supplied with fresh R+ which was replaced every 2-3 days. Cells were allowed to recover until at least day 30 before downstream experiments.

Normal Human Ventricular Cardiac Fibroblasts (NHCF-V) were obtained from Lonza (CC-2904) and cultured according to the manufacturer's instructions. All cells were maintained in a sterile incubator at 37°C with 5% CO_2.

Multi-electrode arrays

iCell Cardiomyocytes were thawed as described above. 48-well BioCircuit microelectrode array (MEA) plates (Axion Biosystems, M768-Bio-48) were coated with sterile-filtered fibronectin bovine plasma (Sigma Aldrich, F1141) prepared in 1x sterile PBS. 5 µL of fibronectin was placed in the center of the electrodes in each well. Water was used to fill the built-in humidity chambers. The fibronectin-coated MEA plate was incubated for 1 hour at 37 °C. Fibronectin was aspirated, and iCell Cardiomyocytes were resuspended in iCell Cardiomyocyte Plating Media (FujiFilm) at a concentration of $1×10^7$ cells per ml and seeded in the MEA plates with 50,000 cells per well. One hour after seeding, 300 µL of

Plating Medium was added to the wells. Media was then exchanged with 300 µL of iCell Cardiomyocyte Maintenance Medium (FujiFilm) and refreshed every 2-3 days.

MEA activity was measured using the AxIS Navigator software (Axion BioSystems, Inc) while docked in a Maestro Multiwell, 768-channel MEA system (Axion BioSystems, Inc). Cells were electrically paced at 1 Hz using the dedicated pacing electrode. Recordings were later batch processed using AxIS Navigator software (Axion BioSystems, Inc) and analyzed using Cardiac Analysis Tool (Axion BioSystems, Inc) to obtain CV and FPD.

Preparation of µEHTs

"Dogbone-shaped" PDMS stencils were prepared as has been previously described (33, 34). Briefly, PDMS stencils consisting of a 1 mm long and 400 mm wide shaft and two 1mm-by-1mm knobs were prepared by pouring Sylgard 184 over a silicone wafer with a positive "dogbone-shaped" mold, which was clamped between glass/acrylic plates and cured at 60°C for at least 8 hours. Stencils were cut, autoclave sterilized, and soaked in methanol for at least 8 hours, and then placed into tissue culture well plates that had been coated with 20 mg/mL fibronectin (Sigma, F1141) at room temperature for at least 8 hours and then briefly rinsed with methanol. Stencils were allowed to attach at 60°C for at least 6 hours. Adhered stencils were then disinfected with two washes in 70% ethanol of at least 8 hours each. After removing ethanol from the second wash, the stencils were allowed to dry completely. Stencils were then coated in 1% sterile Pluronic F68 for 2 hours, followed by three rinses in PBS.

WTC-derived cardiomyocytes were washed twice in PBS and then dissociated by incubating in 0.6 mg/ml Collagenase II in Hank's Balanced Salt Solution at 37°C and 5% CO_2 for 3-4 hours, until cells were easily singularized with gentle pipetting. Collagenase II was quenched in RPMI 1640 with 20% FBS, 1X GlutaMax (Gibco, 35050061), and 1X MEM Non-Essential Amino Acids (Gibco, 11140050), supplemented with 10 µM Y-27632, 150 mg/ml L-ascorbic acid, 4 mg/ml Vitamin B12, and 3.2 mg/ml penicillin (EB20 medium). Cells were then centrifuged at 300 g for 5 minutes. Cells were allowed to recover for 10 minutes in EB20 media at room temperature, then were resuspended with gentle pipetting.

NHCF-V were washed twice with PBS and then dissociated using TrypLE Select enzyme (Gibco, 12604013) for 5 minutes at 37°C and 5% CO_2. The enzyme was quenched by dilution in PBS. NHCF-V were then pelleted by centrifugation at 300 g for 5 minutes and resuspended in EB20.

Cells were counted and viability was confirmed using the Countess 3 automated cell counter (Thermo Fisher). Cells were mixed with gentle pipetting at a ratio of 90% hiPSC-CMs and 10% NHCF-V and centrifuged at 300 g for 5 minutes. Cells were resuspended at a concentration of 70×10^7 cells / mL in EB20 and seeded at 2.1×10^5 cells per stencil. µEHTs were incubated at 37°C and 5% CO_2 for 1 hour and 45 minutes to allow attachment to the dish surface before adding EB20 media to cover tissues. The next day, media was changed to RPMI + B27, with subsequent media changes performed every 2-3 days.

Tissues were allowed to compact for 7 days, after which spontaneous beating and tissue morphology were assessed prior to use for downstream experiments.

Optocardiography

Electrophysiologic characterization of murine hearts and µEHTs was achieved using optical mapping. For µEHTs, the calcium indicator GCaMP6, which uses calcium-binding calmodulin to generate calcium-sensitive fluorescence, is constitutively expressed in the WTC hiPSC-CM line and was used to measure calcium waveform parameters (39). CV and ERP were measured using the potentiometric dye Di-4-ANEPPS (Invitrogen, D1199). A stock solution of Di-4-ANEPPS was prepared at a concentration of 1.25 mg/ml in DMSO, and then diluted to a final concentration of 4.875 mg/ml in RPMI 1640 without phenol red (ThermoFisher, 11835030) + B-27 (Clear R+) immediately prior to use. Media was removed from µEHTs and replaced with Clear R+ with Di-4-ANEPPS for 1 hour and 45 minutes. The Di-4-ANEPPS solution was then removed and replaced with fresh Clear R+. µEHTs were allowed to recover for at least 30 minutes prior to mapping. Di-4-ANEPPS solutions and stained tissues were protected from light throughout.

Both calcium and potentiometric measurements were made with µEHTs in hiPSC-CM Tyrode's solution, which was prepared with 140 mM NaCl, 4.5 mM KCl, 10.3 mM glucose, 10 mM HEPES (Sigma Aldrich, H3375-500G), 1 mM MgCl, and 1.8 mM CaCl at a pH of 7.4. Immediately prior to mapping, media was replaced with hiPSC-CM Tyrode's solution warmed to 37°C. µEHTs were maintained at 37°C during mapping using a heating plate

(Auber Instruments, HP-150). Tissues were electrically stimulated using a PowerLab 15T data acquisition unit (ADInstruments, PL15T02) controlled with LabChart (v7.3.8, ADInstruments). Field stimulation was used for calcium handling measurements, and point stimulation was used when using Di-4-ANEPPS for CV and ERP measurements.

Excitation was performed with an LEX3 LED light source (SciMedia) at 465 nm for GCaMP6 and 530 nm for Di-4-ANEPPS. A DLFLSP2R fluorescent beam splitter with dichroic mirror (SciMedia) equipped for Di-4-ANEPPS (excitation = single-band pass 520/35 nm, emission = long-pass 600 nm) or GCaMP6 (excitation = single-band pass 466/40 nm, emission = single-band pass 525/50 nm) was used for epi-fluorescence and fluorescence detection. Fluorescence was recorded with a MiCAM03-N256 imaging system (SciMedia) equipped with a Leica Plan Apo 1.6x objective and Canon EF Macro 135mm 1:2 lens. Data was collected with the BV Workbench software (v2.7.2., SciMedia).

For both field stimulation and point stimulation, threshold voltages were determined by stimulating μEHTs at 1 Hz at escalating voltages until 1:1 capture was achieved. μEHTs were paced at 1.5x threshold voltage for all subsequent measurements. For calcium handling assessment, tissues were field stimulated at 1 Hz. For CV measurement, point stimulation was performed at 1 Hz. ERP was then determined using a 10 second, 1 Hz S1 drive train followed by shortening S1-S2 interval. The shortest S1-S2 interval at which capture was achieved with the S2 stimulus was defined as the ERP.

Calcium waveform parameters were calculated using custom MATLAB software previously described by Oguntuyo et al. (available at: https://huebschlab.wustl.edu/resources-2/) (75). Di-4-ANEPPS recordings were analyzed and CV measured using MATLAB with Rhythm 3.0, and open-source software, with minor modifications (available at: https://github.com/optocardiography/Rhythm-3.0) (76). Analysis was performed by investigators who were blinded to sample conditions. To ensure rigor and reproducibility, tissues were included in the analysis only if they met the following criteria: 1) one-to-one capture during pacing at 1 Hz; 2) a clear and smooth color gradient from left to right through the shaft on the activation map, indicating organized conduction without conduction block; 3) ≥ 20 vectors generated during analysis; 4) a standard deviation of all vectors used to compute the CV that was ≤ 1/3 of the calculated mean CV.

For quantification of CV in murine hearts, optical mapping was performed similarly, with the following specifications, as has been previously described (15, 76). Briefly, hearts were explanted and Langendorff perfused with murine Tyrode's solution prepared with 128.2 mM NaCl, 4.7 mM KCl, 11.1 mM glucose, 1.19 mM NaH2PO4, 20 mM NaHCO3, 1.05 mM MgCl2, and 1.3 mM CaCl at a pH of 7.4. The solution was oxygenated and maintained at 37°C. Blebbistatin (Cayman Chemical) was added to the perfusate to eliminate motion artifact, and Di-4-ANEPPS was added for potentiometric measurements, after which the hearts were protected from ambient light. Electrical point stimulation was performed at 10 Hz to measure CV. Excitation was performed with a 524 nm light source, emitted fluorescence was filtered with a single long-pass 650 nm filter and recorded by

CMOS camera (BrianVision LLC), and data was collected through the MiCAM Ultima software (v2011.11, BrianVision LLC). Recordings were analyzed and CV measured using MATLAB with Rhythm (Rhythm2014b, available at: https://github.com/optocardiography). Activation maps were generated based on surface coordinate distances and the time of action potential upstroke (defined by maximum voltage derivative, $(dV_m/dt)_{max}$). CV was calculated based on isochrone distances and times of action potential upstroke originating from the stimulation site.

Irradiation

Murine and hiPSC-CM irradiation was performed using the small animal radiation research platform (SARRP, Xstrahl Inc.) as has been previously described (15). Briefly, the SARRP is equipped with an X-ray tube mounted on a gantry and a motorized stage on which anesthetized mice or cell culture plates can be placed. This allows for a cone-beam CT imaging mode (60 kVp, 0.8 mA, 1 mm aluminum filtration) for imaging mouse hearts and a therapy mode (220 kVp, 13 mA, 0.15 mm copper filtration) for radiation delivery to mice or cells (77). Mice were anesthetized using isoflurane. Cone-beam CT was performed, and images were reconstructed and imported into Muriplan (v3.0 Xstrahl Inc.) for treatment planning. The heart was then irradiated using anterior-posterior opposed beams from the 10 mm×10 mm collimator. X-rays were delivered at a 3.62-3.75 Gy/min dose rate to a total dose of 25 Gy in a single fraction. In murine studies, the entire volume of the heart was targeted. Due to differing access to facilities, IR for Seahorse experiments was performed as described below. For hiPSC-CM studies, the entire cell

plate received the indicated dose. In murine studies, littermate sham controls underwent isoflurane anesthesia and CT imaging but no isocentric ionizing radiation. In hiPSC-CM studies, sham control cells from the same cell vial (iCells) or differentiation (WTC) were transported to the irradiator and placed on the benchtop for the duration of IR treatment.

Murine Cardiomyocyte Enrichment by PCM1 Sorting

Cardiomyocyte nuclei isolation was performed as has been previously described (26), with modifications as follows. Snap-frozen or freshly collected murine LVs were diced into 1mm-by-1mm pieces, washed with PBS, and suspended in 10 ml of homogenization buffer (0.32 M Sucrose, 5 mM $CaCl_2$, 3 mM MgAc, 2 mM EDTA pH 8, 0.5 mM EGTA, 10 mM Tris-HCl pH 8, Spermine 0.1 M, and Spermidine trihydrochloride 0.1 M) and supplemented with RNasin Plus (80 units/mL, ProMega N2615) and protease inhibitor (cOmplete Mini EDTA-free protease inhibitor tablet, 1 tab/10 mL buffer, Roche). Tissue was then dounce homogenized for 30 strokes with glass pestle A (loose pestle, Kimble), followed by addition of 20 µL IGEPAL CA-630 detergent buffer (Sigma, I8896) diluted in 80 µL homogenization buffer, and then another 30 strokes with pestle A. Subsequent homogenization with 20 strokes from glass pestle B (tight pestle, Kimble) was performed, followed by trypan blue staining to confirm nuclei extraction. Samples were then strained using a 40 mm nylon cell strainer (Corning) and centrifuged at 1700 g for 8 minutes at 4°C. Supernatant was discarded and the pelleted nuclei were resuspended in 1 mL of supplemented homogenization buffer, followed by staining using anti-PCM1 (1:1000, MilliporeSigma, HPA023370) for 30 minutes on a Nutator shaker at 4°C. Samples were

again centrifuged at 1700 g for 5 minutes, the supernatant discarded, and the pellet resuspended in 1 mL of supplemented homogenization buffer. Secondary staining was performed with Alexa Fluor 647 (goat anti-rabbit, 1:1000, Invitrogen, A21244) and DAPI (1:45000, Millipore Sigma) for 20 minutes on a Nutator shaker at 4°C. Nuclei were again pelleted and resuspended as above, followed by filtering using a 30 mm CellTrics strainer (04-004, 2326 Systmex). A MoFlo sorter (Beckman Coulter) with a 100 mm nozzle at the Siteman Flow Cytometry Core at Washington University was then used to collect DAPI-positive, PCM1-positive populations after size selection and double exclusion. Sample gating was performed for each sample based on single-positive (DAPI-only or AF647-only) and unstained controls. Populations of 2n and 4n nuclei were quantified using FlowJo software (FlowJo LLC).

Isolation of RNA

Total RNA was isolated from hiPSC-CM monolayers using RNeasy Micro Kit (Qiagen) according to the manufacturer's instructions, including treatment with RNase-free DNase (Qiagen). Total RNA was isolated from murine ventricles using TRIzol reagent (Invitrogen), per manufacturer instructions, and DNase-treated using the TURBO DNA-free Kit (Ambion).

RNA-sequencing

Total RNA was isolated as described above from iCells at 1, 7, and 14 days after IR, and from GCaMP6 hiPSC-CMs at 1, 7, 14, and 28 days after IR. Quantification and quality assessment was performed using a 2100 Bioanalyzer (Agilent). RNA-seq was then performed by the Genome Technology Access Center at Washington University School of Medicine. Briefly, samples were prepared according to library kit manufacturer's protocol, indexed, pooled, and sequenced on an Illumina NovaSeq 6000. Basecalls and demultiplexing were performed with Illumina's bcl2fastq2 software. RNA-seq reads were then aligned and quantitated to the Ensembl release 101 primary assembly with an Illumina DRAGEN Bio-IT on-premise server running version 3.9.3-8 software.

ATAC-sequencing

ATAC-seq was performed per the manufacturer's instructions (Active Motif, 53150) with minor modifications (78). Briefly, adherent iCell or WTC hiPSC-CM monolayers were dissociated at the specified timepoints using 0.05% trypsin for 5 minutes, reactions were quenched in media, cells were pelleted and then washed in ice cold PBS. 2.0×10^5 cells were used per sample. For murine experiments, 1.0×10^5 $PCM1^+$ nuclei were isolated as described above. Cells or nuclei were lysed in ATAC-seq lysis buffer. Samples were then processed for tagmentation and DNA was purified, followed by PCR amplification for 10 cycles using Nextera Multiplex Primers (Active Motif, 53155). Double-sided bead purification was performed using AMPure XP SPRI Reagent (Beckman Coulter, A63880) to remove primer dimer and large DNA fragments. Samples were quantified by Qubit and analyzed using Agilent BioAnalyzer to confirm library quality. Samples were sequenced

on a NovaSeq 6000 targeting 50 million reads/library at the Washington University the Genome Technology Access Center.

Cleavage Under Targets and Tagmentation (CUT&Tag)

CUT&Tag was performed per the manufacturer's instructions (Active Motif, 53160) with minor modifications (79). Briefly, cells were harvested as described above for ATAC-seq. Concavalin A beads were prepared and incubated with ~2.5×10^5 cells for 10 minutes at room temperature with constant mixing. Samples were then incubated at 4°C overnight with constant mixing with 1 ml of the following rabbit primary antibodies: H3K27me3 (Active Motif, 39155), H3K27ac (Active Motif, 39685), H3K4me3 (Active Motif, 39159), H3K4me1 (Cell Signaling Technology, 5326), or IgG isotype control (Cell Signaling Technology, 66362S). Secondary antibody binding with guinea pig anti-rabbit antibody (1:100 dilution) was then performed for 1 hour at room temperature. pA-Tn5 transposomes were allowed to bind for 1 hour at room temperature with constant mixing, followed by tagmentation at 37°C for 1 hour. The tagmentation reaction was then terminated through addition of EDTA, SDS, and proteinase K at 55°C for 1 hour. DNA was purified, PCR amplified, and underwent bead cleanup and sequencing as described above for ATAC-seq.

RNA-seq data processing

Raw RNA-seq reads were processed using a standardized RNA-seq pipeline, which includes data preprocessing, quality control, integrative analysis, and data visualization. Reads were aligned to either the human genome (hg38) or mouse genome (mm10) using STAR (version 2.5.4b) (80). Gene counts were quantified using Subread:featureCounts (version 1.4.6) (81), based on the number of uniquely aligned, unambiguous reads. Gene annotation was performed using GENCODE M20 for mm10 or GENCODE V27 for hg38 (82). To ensure sample integrity, sex labels were verified by examining the expression of sex-biased genes—Xist/XIST (female-specific) and Uty/UTY (male-specific). Only samples with gene expression patterns consistent with their assigned sex labels were retained for downstream analyses.

To remove unwanted variation in gene counts, we applied the RUVr function from the RUVSeq normalization package (83), using k = 3 as the estimated number of unwanted variation factors for both mouse and human samples. Normalized gene counts were then analyzed using DESeq2 (84) to identify differentially expressed genes (DEGs) between sham-treated and radiation-exposed conditions. Genes with CPM > 1.0 were incorporated into a DESeq2 dataset and underwent regularized log transformation (rlog function, DESeq2) for variance stabilization. Statistical significance was determined using the Benjamini-Hochberg correction for false discovery rate (FDR), with adjusted p-values computed via the R stats function p.adjust. DEGs were defined based on the following criteria: absolute log2(fold change) > 1 and adjusted p-value < 0.01. To minimize false positives, genes exhibiting significant expression changes (absolute log2(fold change) > 1 and adjusted p-value < 0.01) under normal culture conditions (pair-wised comparison

between any two culture timepoints) were filtered out. Lists of sham condition versus post-radiation DEGs can be found in Supplemental Tables 5 and 6. The k-means clustering was used to detect expression patterns during the cell recovery process. The number of clusters (k) was determined by examining the within-groups sum of square error (SSE), when the SSE decreased to less than 5%. The Gene Ontology (GO) analysis and pathway analysis of DEGs were performed by using ToppFun (85).

CUT&Tag data processing

CUT&Tag data were processed as described in a previous study (86). Briefly, raw FASTQ reads from each biological replicate were quality-trimmed using Cutadapt (--quality-cutoff 15,10) to remove low-quality ends and then aligned to the hg38 reference genome using BWA (87). The resulting BAM files were processed using methylQA in density mode without read extension (-E 0) to represent fragments accurately. Enriched histone peaks were identified separately for each epigenetic modification using the callpeak function of MACS2 (88) (-f BEDPE --nomodel --shift -100 --extsize 200 --broad), with input samples as controls and a q-value cutoff of 0.1. To ensure consistency across biological replicates, peak files for each epigenetic marker were merged using the merge function from the BEDTools suite. Peaks were further filtered based on the following criteria: 1) length > 400 bp; 2) CPM > 5 (mean across all biological replicates); 3) no overlap with the ENCODE blacklist. For differential analysis, unwanted variation was removed using the RUVr function from the RUVSeq normalization package (k = 2). Sham-specific and post-radiation-specific differentially modified regions (DMRs) were identified between day 7

and day 14 samples for each epigenetic marker using edgeR (89), with the following thresholds: FDR < 0.05 & absolute log2(fold change) > log2(1.25).

ATAC-seq data processing

Raw ATAC-seq reads were aligned to the human (hg38) or mouse (mm10) reference genome and processed using AIAP (90), which includes four key steps: data processing, quality control, integrative analysis, and data visualization. To generate a unified peak set, narrow peak files from all ATAC-seq libraries were merged using the merge function in the BEDTools suite (91). Read counts for each ATAC-seq peak were quantified using the BEDTools coverage command. High-quality libraries were selected for downstream analysis based on the following criteria: 1) Reads Under Peak (RUP) ratio > 5%; 2) coding enrichment ratio > 2; 3) total reads > 30 million.

To correct batch effects in read counts, RUVr normalization (RUVSeq R package, k = 2) was applied. Differentially accessible regions (DARs) specific to sham and post-radiation conditions were identified using edgeR, with the following thresholds defined in a previous study (92): FDR < 0.05 & absolute log2(fold change) > log2(1.5). The k-means clustering was used to detect expression patterns during the cell recovery process as previous study (93). The number of clusters (k) was determined by examining the within-groups sum of square error (SSE), when the SSE decreased to less than 5%. Functional enrichment analysis of genes near sham-specific and post-radiation-specific DARs was performed using the GREAT tool (v4.0.4) (94) to identify associated GO terms and pathways.

Motif analysis

HOMER (v4.11.1) (95) was used to calculate the motif enrichment and genomic enrichment under the sham-specific or post-radiation-specific DMRs and DARs. The motifs with the match score should be at least 0.85, the p-value cutoff as 1e-11, and at least presented in >5% DMRs or DARs are considered.

Visualizing deeply sequenced data by metaplot and heatmap plots

ATAC-seq or CUT&Tag signals on sham-specific or post-radiation-specific regions were calculated by using deepTools (96) with parameter detailed as “computeMatrix reference-point—referencePoint center -a 5000 -b 5000 -bs 100 –missingDataAsZero”. Subsequently, averaged ATAC-seq and H3K27ac signals across these regions were visualized using the plotHeatmap function in the deepTools package, allowing for a direct comparison of chromatin accessibility and histone modification patterns between sham and post-radiation conditions.

Protein Extraction

Cellular fractionation and isolation of membrane protein was performed in hiPSC-CMs using the Mem-PER™ Plus Membrane Protein Extraction Kit (Thermo Fisher) per the manufacturer’s instructions. Briefly, cells were scraped into media and centrifuged at 500

g for 5 minutes, followed by two washes in the provided Cell Wash Solution. Cells were resuspended in the provided Cell Permeabilization Buffer supplemented with Halt™ Protease and Phosphatase Inhibitor Cocktail (Thermo Fisher Scientific, 78440), followed by incubation at 4°C with constant mixing for 10 minutes. Samples were then centrifuged at 16,000 g for 15 minutes at 4°C. The supernatant was transferred and stored as the cytoplasmic fraction. Pellets were then resuspended in the provided Membrane Solubilization Buffer, followed by incubation at 4°C with constant mixing for 30 minutes. Samples were centrifuged as above, and the supernatant was saved as the membrane fraction.

For whole cell lysate preparation, media was removed and cells were washed twice in PBS. Cells were then incubated for 5 minutes on ice with RIPA lysis buffer (50 mM Tris-HCl, 150 mM NaCl, 1% Triton X100, 0.50% sodium deoxycholate, 0.1% SDS, 5 mM EDTA) supplemented with Halt™ Protease Inhibitor Cocktail (Thermo Fisher Scientific, 78430). Cells were then homogenized by scraping and placed for 60 minutes on a Nutator shaker at 4°C. Samples were centrifuged for 10 minutes at 13200 g at 4°C and supernatant was saved and aliquoted.

Protein concentration was measured using a commercial BCA assay prior to western blotting (Thermo Fisher). Samples were stored at -80°C for future use. For membrane fractionated samples, successful fractionation was confirmed using immunoblotting (performed as described below) to identify the isolation of GAPDH in the cytoplasmic fraction and Na/K ATPase in the membrane fraction.

Western Blotting

Gel electrophoresis was performed using pre-cast 4-15 or 4-20% Tris-Glycine gels (Biorad). Proteins were then transferred by wet transfer overnight to polyvinylidene difluoride (Millipore Sigma) membranes. Blots were blocked for 1 hour in milk blocking buffer (5% milk in Tris-buffered saline, 0.05% Tween-20) or 5 minutes in EveryBlot Blocking Buffer (Biorad). Immunoblotting was performed overnight at 4 °C for all antibodies at the following dilutions: anti-$Na_V1.5$ 1:1000 (Cell Signaling Technologies, 14421), anti-Na/K ATPase 1:500 (Cell Signaling Technologies, 23565), anti-Connexin43 1:1000 (Sigma, C6219), anti-ATM 1:1000 (Cell Signaling Technologies, 2873), anti-phospho-ATM 1:1000 (Ser1981, Cell Signaling Technologies, 13050), anti-DNA-PK 1:1000 (Cell Signaling Technologies, 38168), anti-phospho-DNA-PK 1:1000 (Ser2056, Cell Signaling Technologies, 68716), and anti-GAPDH 1:2000 (Cell Signaling Technologies, 2118). Secondary detection was performed using horseradish peroxidase-conjugated antibodies at a 1:5000 dilution (Abcam, ab6721) and Clarity Western ECL Substrate (BioRad). Densitometry analysis was performed using ImageJ and normalized to Na/K ATPase density.

Histology and Immunofluorescence

Human heart tissue was fixed in 4% PFA and paraffin embedded. H&E staining was performed on 5 mm sections per the manufacturer's instructions (Abcam, ab245880).

Mouse hearts and μEHTs were arrested in diastole using 50 mM KCl, followed by fixation with PFA and embedding in paraffin. Samples were sectioned at a thickness of 5 mm for immunohistochemistry. For hiPSC-CMs, fixation was performed with 2% PFA for 10 minutes at room temperature, followed immediately by immunocytochemistry. Permeabilization was performed with 0.2% Triton X100 (Invitrogen, HFH10). Blocking was performed using ReadyProbes 2.5% Normal Goat Serum (Invitrogen, R37624). Primary antibodies were incubated at 4°C overnight after being diluted in blocking solution as follows: N-Cadherin 1:100 (Cell Signaling Technology, 13116), PCM1 1:200 (MilliporeSigma, HPA02337), and rabbit anti-vimentin 1:50 (Cell signaling technology 5741). Secondary antibodies were diluted 1:200 in blocking solution and incubated for 1 hour at room temperature. Wheat germ agglutinin (WGA) (ThermoFisher Scientific, W56133) staining was performed at a dilution of 2.5 μL/ml for 30 minutes at room temperature. Cells were stained with DAPI (ACD Biotechne, 320858) and mounted with Vectashield Antifade Mounting Media (Vector Laboratories, H-1000-10). For quantification of fibrosis, mouse heart sections were stained with Masson's trichrome (StatLab, KTMTR) according to the manufacturer's instructions. Imaging was performed with a confocal microscope (Zeiss) or Zeiss Axio 7 Brightfield Slide Scanner while maintaining identical settings for each immunohistochemical target. Analysis was performed with ImageJ/Fiji (v 2.14.0/1.54f).

Measurement of OCR in hiPSC-CMs

Mitochondrial respiration was assessed using the Seahorse XFe96 Extracellular Flux Analyzer in conjunction with the XF Cell Mito Stress Test Kit, following the manufacturer's guidelines (Agilent Technologies, Santa Clara, CA). In brief, human-induced pluripotent stem cell-derived cardiomyocytes (hiPSC-CMs) were seeded onto a XF96 cell culture microplate before being exposed to varying single radiation doses using an RS2000 160kV X-ray Irradiator using a 0.3-mm copper filter (Rad Source Technologies). On the day of the experiment, cells were washed with pre-warmed XF Assay Medium and incubated in a non-CO_2 incubator at 37˚C for 1 hour in XF Assay Medium. For the assay, cells were sequentially treated with the following final concentrations: 2.5 μM oligomycin, 2 μM FCCP (carbonyl cyanide-p-trifluoromethoxyphenylhydrazone), and 1 μM rotenone plus 1 μM antimycin A. Data collection and analysis were performed using the Seahorse Wave software (RRID: SCR_014526). All reagents were obtained from Agilent Technologies.

Flow cytometric detection of ROS

iCells were plated as above and maintained in maintenance medium for 5 days. Cells were then either sham-treated or exposed to 25 Gy IR. To assess total or mitochondrial ROS, cells were incubated with CellROX (Thermo Fisher C10492) or MitoSOX (Thermo Fisher M36008) at a final concentration of 2.5 μM for 30 min at 37°C, respectively. Flow cytometric analysis was performed 24 hr or 7 days post-IR. The proportion of ROS-positive cells was determined using standard gating strategies.

Supplemental References

71. Sohal DS, et al. Temporally regulated and tissue-specific gene manipulations in the adult and embryonic heart using a tamoxifen-inducible Cre protein. *Circ Res*. 2001;89(1):20–25.

72. Marino S, et al. Induction of medulloblastomas in p53-null mutant mice by somatic inactivation of Rb in the external granular layer cells of the cerebellum. *Genes Dev*. 2000;14(8):994–1004.

73. Lian X, et al. Directed cardiomyocyte differentiation from human pluripotent stem cells by modulating Wnt/β-catenin signaling under fully defined conditions. *Nat Protoc*. 2013;8(1):162–175.

74. Tohyama S, et al. Distinct Metabolic Flow Enables Large-Scale Purification of Mouse and Human Pluripotent Stem Cell-Derived Cardiomyocytes. *Cell Stem Cell*. 2013;12(1):127–137.

75. Oguntuyo K, et al. Robust, Automated Analysis of Electrophysiology in Induced Pluripotent Stem Cell-Derived Micro-Heart Muscle for Drug Toxicity. *Tissue Eng Part C Methods*. 2022;28(9):457–468.

76. Laughner JI, et al. Processing and analysis of cardiac optical mapping data obtained with potentiometric dyes. *American Journal of Physiology-Heart and Circulatory Physiology*. 2012;303(7):H753–H765.

77. Wong J, et al. High-Resolution, Small Animal Radiation Research Platform With X-Ray Tomographic Guidance Capabilities. *International Journal of Radiation Oncology*Biology*Physics*. 2008;71(5):1591–1599.

78. Buenrostro JD, et al. Transposition of native chromatin for fast and sensitive epigenomic profiling of open chromatin, DNA-binding proteins and nucleosome position. *Nat Methods*. 2013;10(12):1213–1218.

79. Kaya-Okur HS, et al. CUT&Tag for efficient epigenomic profiling of small samples and single cells. *Nature Communications*. 2019;10:1930.

80. Dobin A, et al. STAR: ultrafast universal RNA-seq aligner. *Bioinformatics*. 2013;29(1):15–21.

81. Liao Y, Smyth GK, Shi W. The Subread aligner: fast, accurate and scalable read mapping by seed-and-vote. *Nucleic Acids Res*. 2013;41(10):e108.

82. Harrow J, et al. GENCODE: the reference human genome annotation for The ENCODE Project. *Genome Res*. 2012;22(9):1760–1774.

83. Risso D, et al. Normalization of RNA-seq data using factor analysis of control genes or samples. *Nat Biotechnol*. 2014;32(9):896–902.

84. Love MI, Huber W, Anders S. Moderated estimation of fold change and dispersion for RNA-seq data with DESeq2. *Genome Biol*. 2014;15(12):550.

85. Chen J, et al. ToppGene Suite for gene list enrichment analysis and candidate gene prioritization. *Nucleic Acids Res*. 2009;37(Web Server issue):W305-311.

86. Cheng S, et al. Review and Evaluate the Bioinformatics Analysis Strategies of ATAC-seq and CUT&Tag Data. *Genomics Proteomics Bioinformatics*. 2024;22(3):qzae054.

87. Li H, Durbin R. Fast and accurate short read alignment with Burrows-Wheeler transform. *Bioinformatics*. 2009;25(14):1754–1760.

88. Zhang Y, et al. Model-based analysis of ChIP-Seq (MACS). *Genome Biol*. 2008;9(9):R137.

89. Robinson MD, McCarthy DJ, Smyth GK. edgeR: a Bioconductor package for differential expression analysis of digital gene expression data. *Bioinformatics*. 2010;26(1):139–140.

90. Liu S, et al. AIAP: A Quality Control and Integrative Analysis Package to Improve ATAC-seq Data Analysis. *Genomics Proteomics Bioinformatics*. 2021;19(4):641–651.

91. Quinlan AR, Hall IM. BEDTools: a flexible suite of utilities for comparing genomic features. *Bioinformatics*. 2010;26(6):841–842.

92. Gontarz P, et al. Comparison of differential accessibility analysis strategies for ATAC-seq data. *Sci Rep*. 2020;10(1):10150.

93. Meganathan K, et al. Regulatory networks specifying cortical interneurons from human embryonic stem cells reveal roles for CHD2 in interneuron development. *Proc Natl Acad Sci U S A*. 2017;114(52):E11180–E11189.

94. McLean CY, et al. GREAT improves functional interpretation of cis-regulatory regions. *Nat Biotechnol*. 2010;28(5):495–501.

95. Heinz S, et al. Simple combinations of lineage-determining transcription factors prime cis-regulatory elements required for macrophage and B cell identities. *Mol Cell*. 2010;38(4):576–589.

96. Ramírez F, et al. deepTools: a flexible platform for exploring deep-sequencing data. *Nucleic Acids Res*. 2014;42(Web Server issue):W187-191.

Supplemental Figures

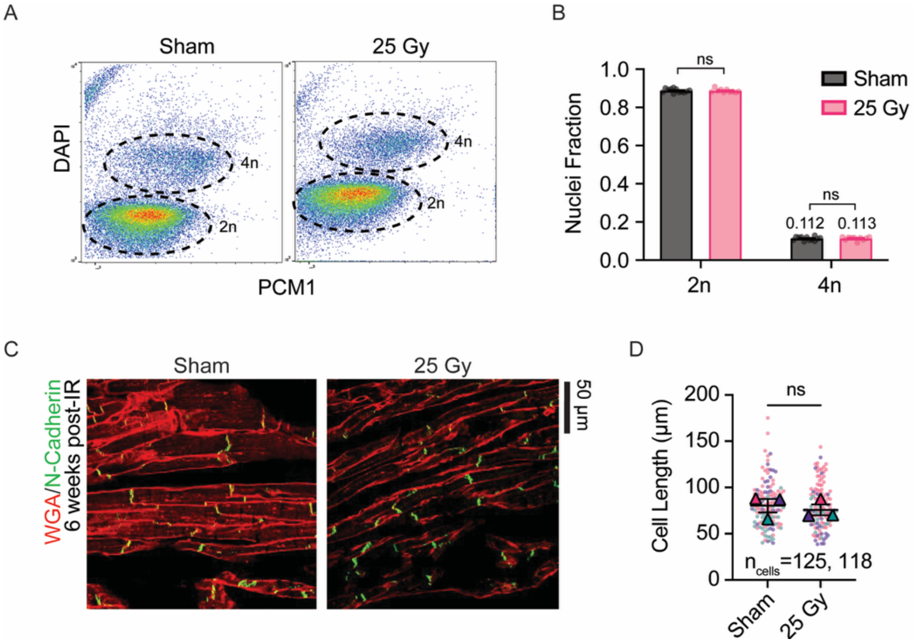

Supplemental Figure 1: Changes to cardiomyocyte nuclear size after 25 Gy are not associated with increased ploidy or cell size. (A) Representative distribution of 2n (lower DAPI) versus 4n (higher DAPI) nuclei in the PCM1-positive population in sham versus irradiated ventricles. **(B)** Nuclear ploidy distribution of sham (grey) versus irradiated (red) PCM1-sorted nuclei (n_{sham} = 12, n_{IR} =10 biologically independent replicates; two-tailed t-test, P = 0.92). **(C)** Representative images of sham versus irradiated mouse hearts showing WGA (red) and N-Cadherin immunostaining (green) at 6 weeks. Scale bare = 50 um. **(D)** Cellular length in sham versus irradiated hearts 6 weeks post-IR (two-tailed t-test, P = 0.79). Colors represent separate mice.

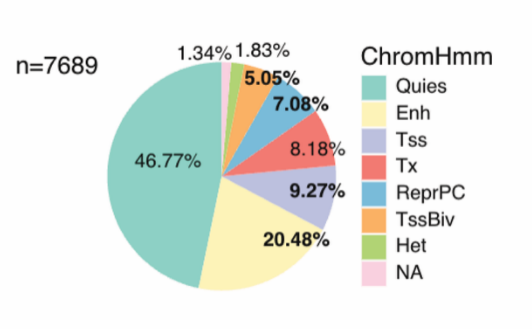

Supplemental Figure 2: **Post-IR PCM1+ nuclei display most prominent changes in chromatin accessibility at quiescent and enhancer chromatin regions.** ChromHMM analysis showing predicted chromatin states for DARs from post-IR murine cardiomyocyte nuclei. Quies = quiescent, Enh = enhancer, TSS = transcription start site, Tx = transcription-related state, ReproPC = repressed PolyComb, TSSBiv = bivalent/poised TSS, Het = heterochromatin, NA = not assigned.

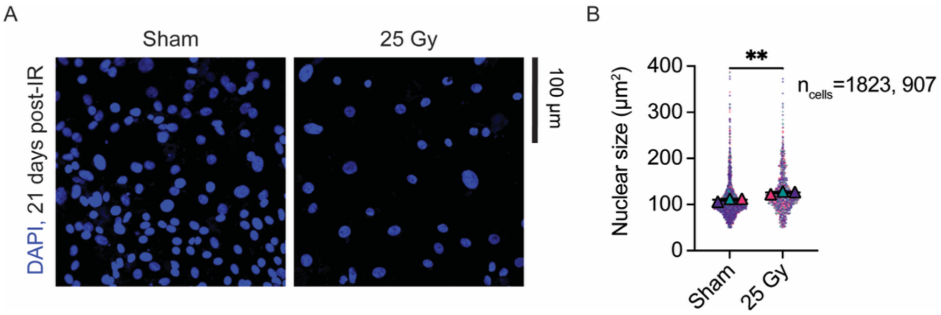

Supplemental Figure 3: 25 Gy increases nuclear size in hiPSC-CMs. (A) Representative fluorescence images of DAPI stained iCells 21 days after sham (left) or 25 Gy (right). Scale bar = 100 µm. **(B)** Quantification of nuclear size in sham versus 25 Gy irradiated hiPSC-CMs (three independent experiments, two-tailed t-test **P = 0.0059). Colors represent biological replicates (distinct cell vials).

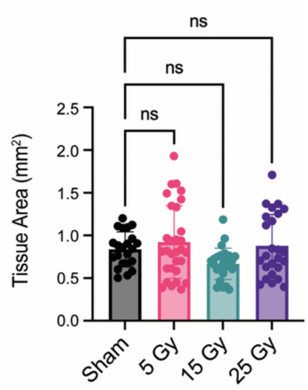

Supplemental Figure 4: **µEHTs size is unchanged after IR.** Quantification of total tissue area of µEHTs 2 weeks after sham, 5 Gy, 15 Gy, or 25 Gy IR (≥22 µEHTs per condition from 5 differentiations; one-way ANOVA: $P = 0.023$; Dunnett's post-hoc test: $P_{adj,sham\text{-}5gy} = 0.66$, $P_{adj,sham\text{-}15gy} = 0.16$, $P_{adj,sham\text{-}25gy} = 0.95$).

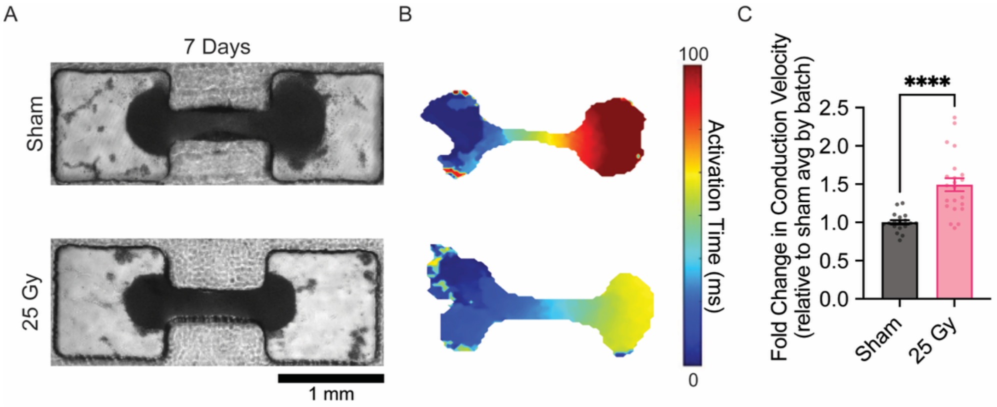

Supplemental Figure 5: **25 Gy induces increased CV in μEHTs within 1 week. (A)** Representative tissue culture images of sham and 25-Gy-irradiated μEHTs 7 days post-treatment. Scale bar = 1mm. **(B)** Representative activation maps from optocardiography of sham and 25-Gy-irradiated μEHTs 7 days after treatment. Tissues were stimulated at 1 Hz on the left knob. **(C)** Quantified CV in sham and 25-Gy-irradiated μEHTs 7 days after treatment, shown as FC relative to sham average by batch (≥18 μEHTs from 3 independent differentiations; two-tailed t-test, **** $P < 0.0001$).

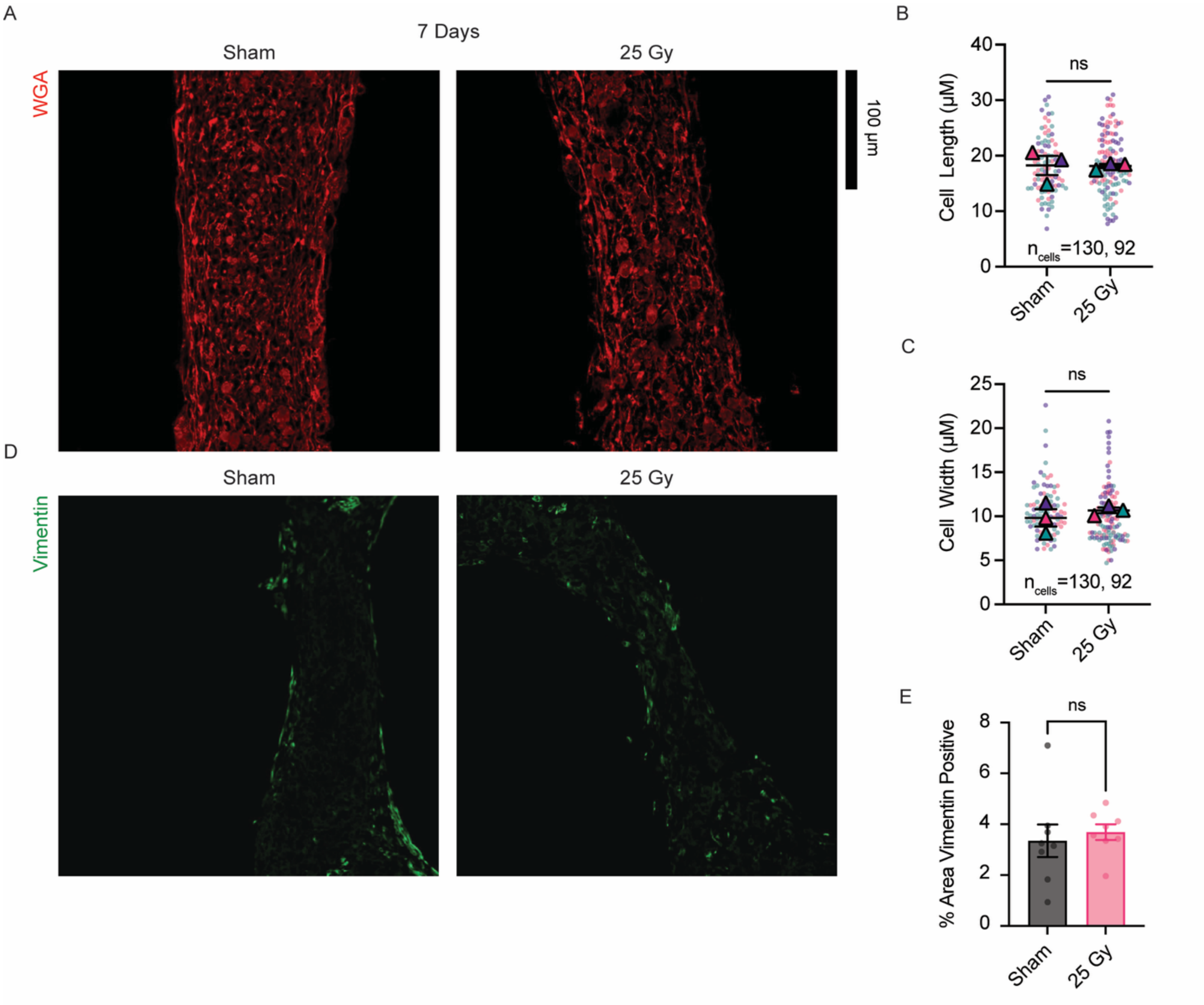

Supplemental Figure 6: Changes in cell size or fibroblast numbers do not explain increased CV in hiPSC-CMs after IR. (A) Immunostaining for WGA in µEHTs 1 week after 25 Gy. Scale bar = 100 µm. **(B)** Quantification of cell length in µEHTs 1 week post-IR compared to sham tissues (3 independent differentiations; two-tailed t-test: P = 0.96). Colors represent biological replicates (distinct differentiations). **(C)** Quantification of cell width in µEHTs 1 week post-IR compared to sham tissues (3 independent differentiations; two-tailed t-test: P = 0.49). Colors represent biological replicates (distinct differentiations). **(D)** Immunostaining for vimentin in µEHTs 1 week after 25 Gy. **(E)** Quantification of the percent area staining positive for vimentin within sham versus

25-Gy-irradiated μEHTs (n = 8 μEHTs per condition from 3 independent differentiations; two-tailed t-test: $P = 0.64$).

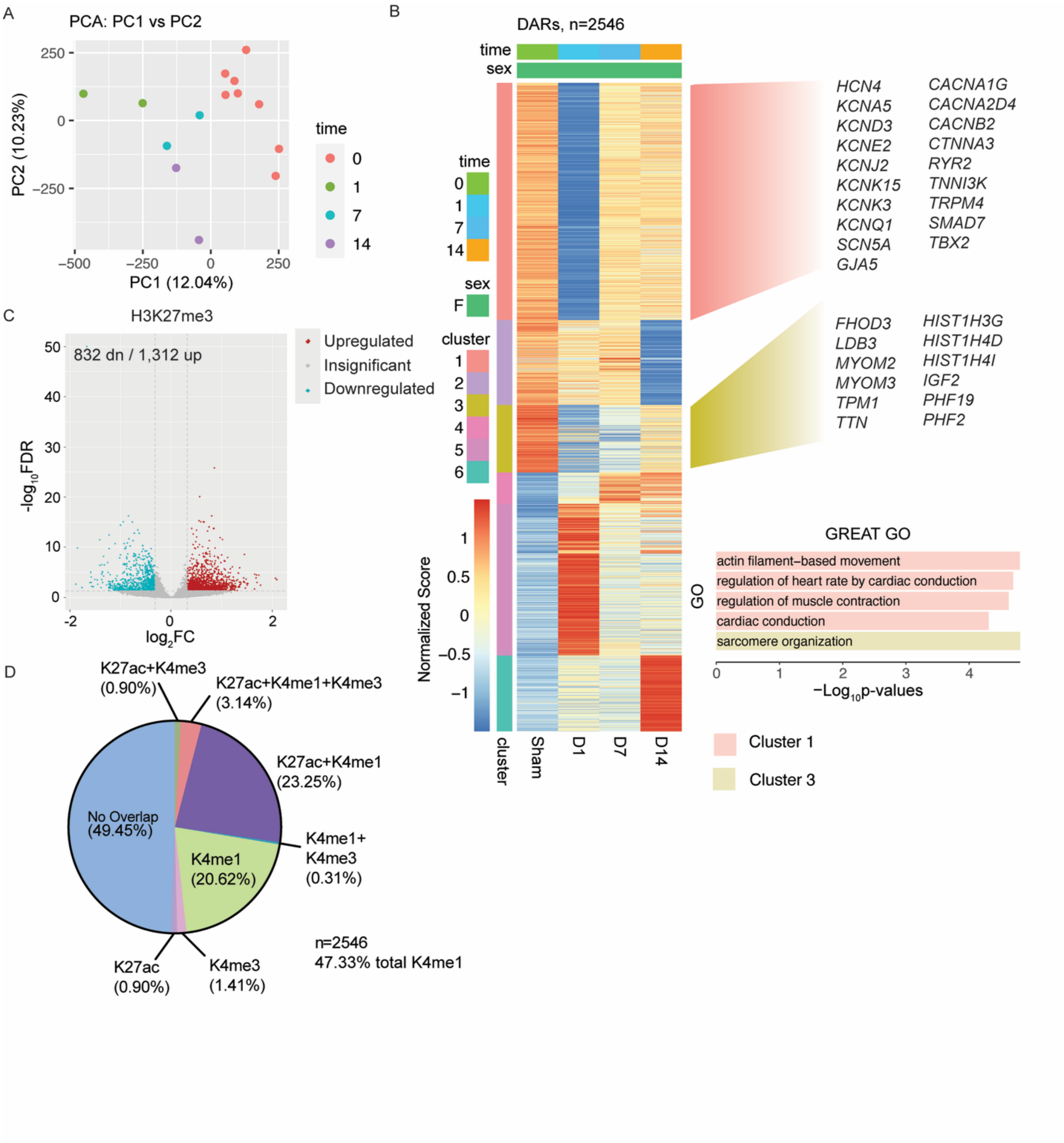

Supplemental Figure 7: 25 Gy induces dynamic changes in chromatin accessibility in iCell hiPSC-CMs. (**A**) PCA of ATAC-seq samples for sham, 1-, 7-, and 14-day post-IR iCells. **(B)** Heat map of DARs in iCells over time after 25 Gy compared to sham organized by K-means clustering with selected enriched GO terms from clusters 1 and 3. Selected genes relevant to cardiac physiology and associated with

DARs driving GO term enrichment are listed. **(C)** Volcano plot for increasing and decreasing DMRs from H3K27me3 CUT&Tag. **(D)** Proportion of DARs labeled by different histone markers from CUT&Tag.

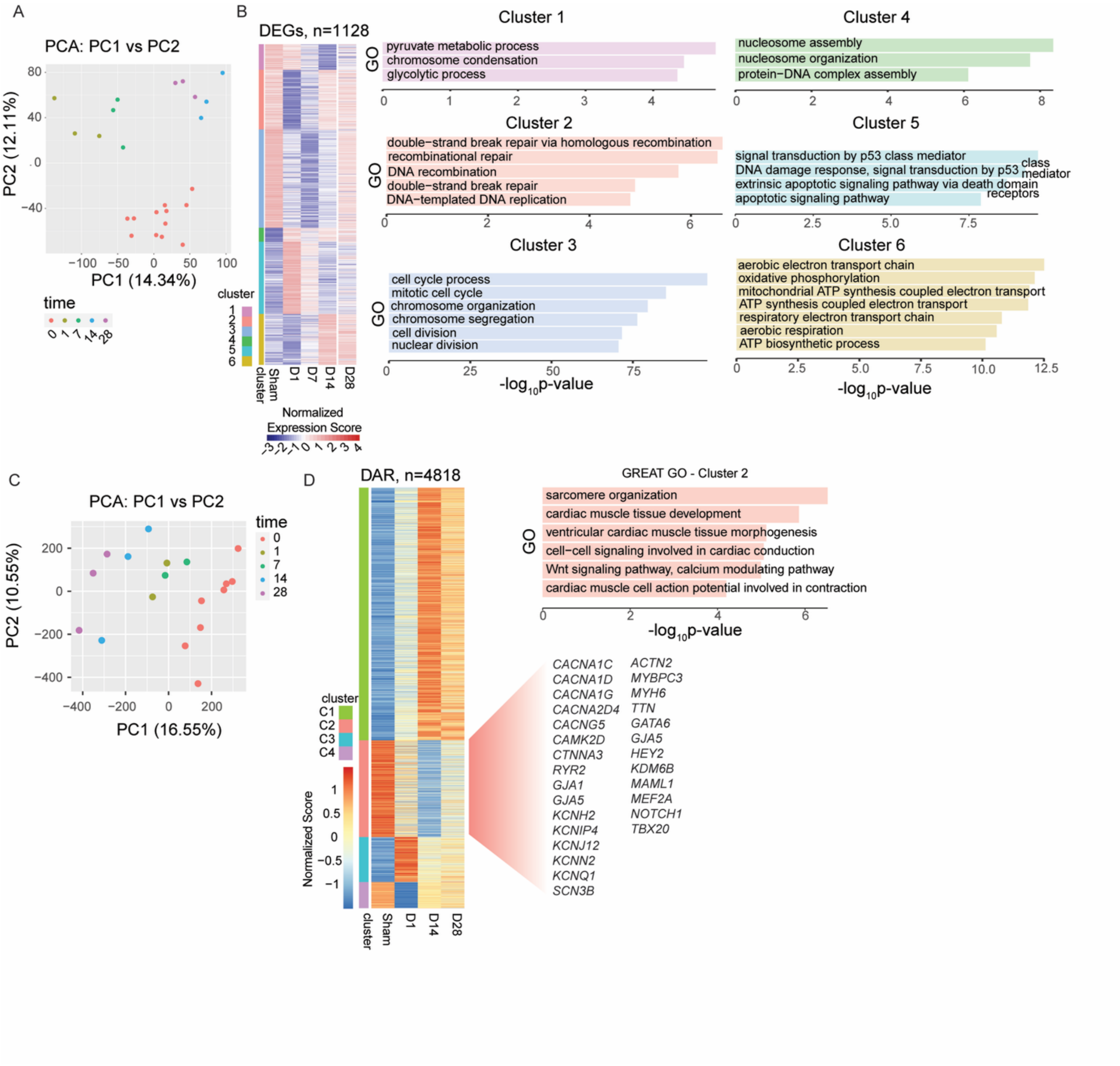

Supplemental Figure 8: Post-IR WTC hiPSC-CMs have distinct transcriptional and chromatin states. (**A**) PCA analysis of RNA-seq samples for sham, 1-, 7-, 14-, and 28-day post-IR GCaMP hiPSC-CMs. **(B)** Heat map of DEGs in GCaMP hiPSC-CMs over time after 25 Gy compared to sham organized by K-means clustering with GO term enrichment by cluster. (**C**) PCA of ATAC-seq samples for sham, 1-, 7-, 14-, and 28-day post-IR GCaMP hiPSC-CMs. **(D)** Heat map of DEGs in GCaMP hiPSC-CMs over time after 25 Gy compared to sham organized by K-means clustering with selected enriched

GO terms from cluster 2. Selected genes relevant to cardiac physiology and associated with DARs driving GO term enrichment are listed.

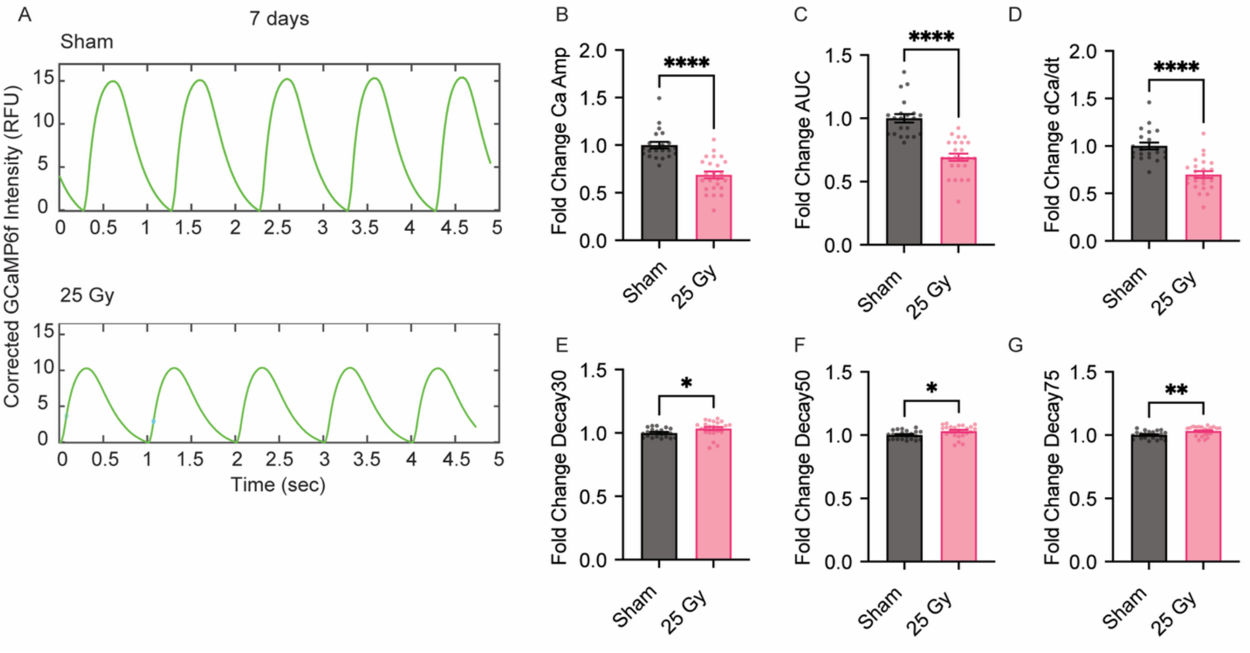

Supplemental Figure 9: 25 Gy induces acute change in calcium handling by 1 week in µEHTs. (A) Representative traces of GCaMP6 fluorescence intensity in µEHTs paced at 1 Hz at a 1-week timepoint after sham or 25 Gy. **(B)** Quantification of calcium handling parameters (Ca Amp = Calcium Amplitude, **F**; AUC, **G**; dCa/dt=change in GCaMP fluorescence / time, **H**; Decay30, **I**; Decay50, **J**; Decay75, **K**) by GCaMP6 fluorescence in sham versus 25-Gy-irradiated µEHTs 7 days after treatment, presented as FC relative to sham average by batch (≥21 µEHTs per condition from 3 independent differentiations; two-tailed t-test: $P_{CaAmp} < 0.0001$, $P_{AUC} < 0.0001$, $P_{dCa/dt} < 0.0001$, $P_{Decay30} = 0.029$, $P_{Decay50} = 0.021$, $P_{Decay75} = 0.0034$).

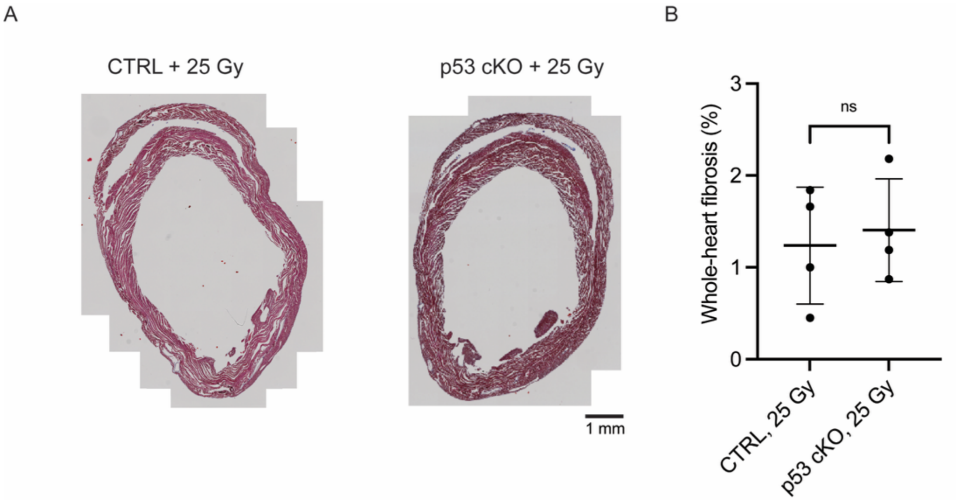

Supplemental Figure 10: Cardiomyocyte-specific p53 knockout does not predispose to increased fibrosis after 25 Gy IR. (**A**) Representative Masson's trichrome staining in control and p53 cKO mice 6 weeks after 25 Gy. Scale bar = 1 mm. (**B**) Quantification of scar size (±SD) as percent of total heart area (n = 4 mice per condition; two-tailed t-test, P = 0.71).

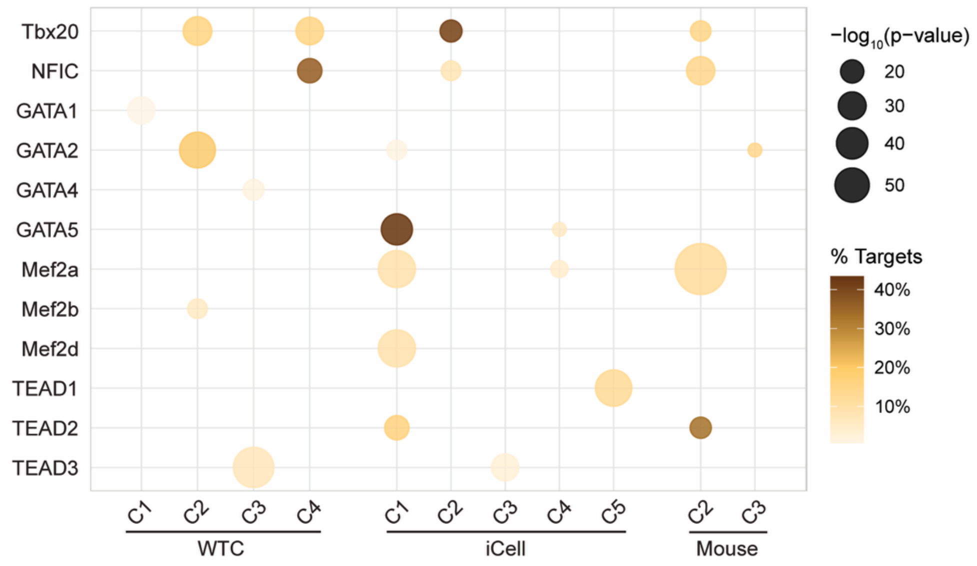

Supplemental Figure 11: Identification of candidate transcriptional regulators of the cardiomyocyte response to IR. Selected HOMER Motif analysis of TF binding motifs enriched in DARs across model systems.
