# Supplementary material for: Cardiac radiotherapy–induced epigenetic memory underlies electrophysiologic and metabolic reprogramming
Source: J Clin Invest. 2026 Feb 17;136(7):e193087. doi: 10.1172/JCI193087 (PMC13038212; doi:10.1172/JCI193087)

Full unedited blots for Figure 3C

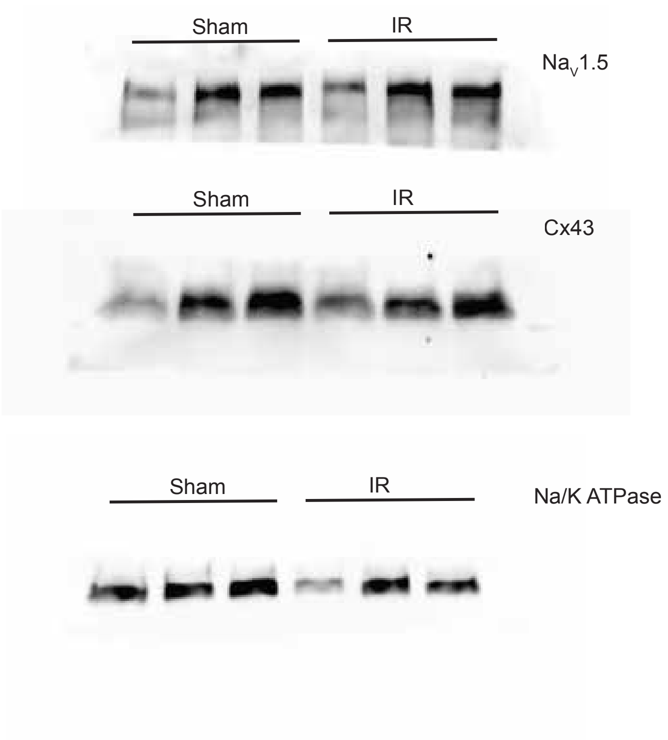

Additional blots quantified for Figure 3D-E

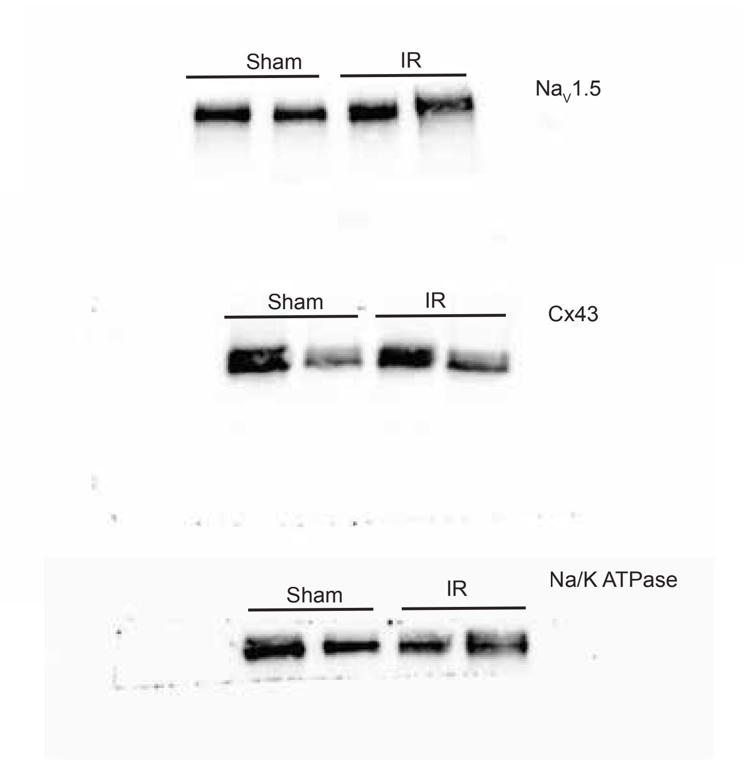

Full unedited blots for Figure 7C, top

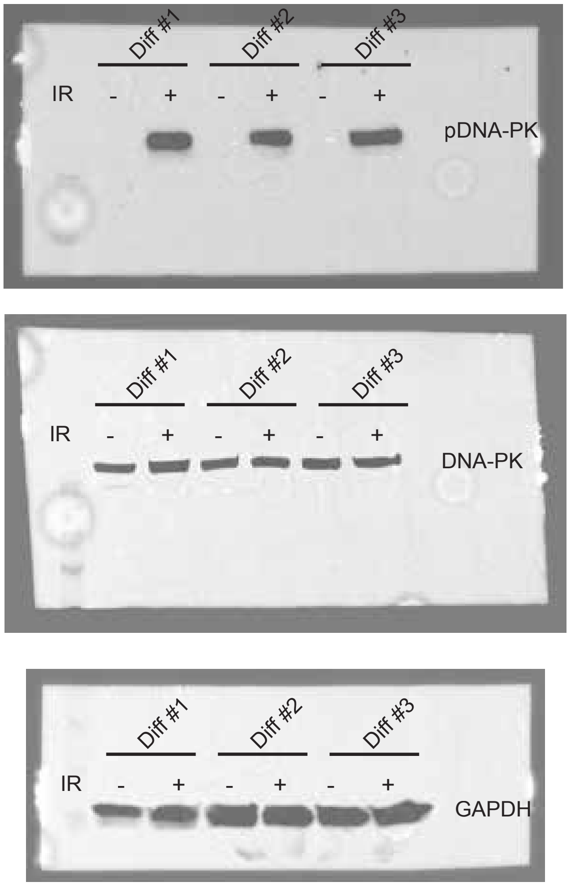

Full unedited blots for Figure 7C, bottom

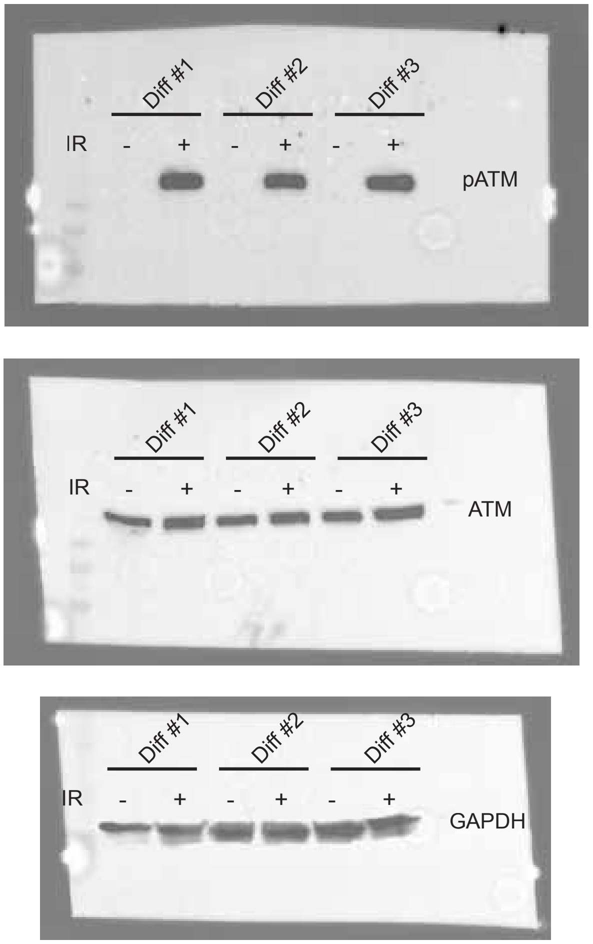

Supplement: Unedited blot and gel images [file jci-136-193087-s009.pdf]
